# 2D to 3D: Exploring Variation of Niche Dimensionality Across Consumers in a Coastal Arctic Ecosystem and Implications on Interpretation

**DOI:** 10.1002/ece3.73671

**Published:** 2026-05-13

**Authors:** Paloma C. Carvalho, Kelsey F. Johnson, Kyle H. Elliott, Steven H. Ferguson, Aaron T. Fisk, H. Grant Gilchrist, Kevin J. Hedges, Oliver P. Love, C. J. Mundy, Andrea Niemi, Wesley R. Ogloff, Bruno Rosenberg, Cortney A. Watt, David J. Yurkowski

**Affiliations:** ^1^ Fisheries and Oceans Canada Winnipeg Manitoba Canada; ^2^ McGill University Ste. Anne de Bellevue Quebec Canada; ^3^ University of Manitoba Winnipeg Manitoba Canada; ^4^ University of Windsor Windsor Ontario Canada; ^5^ Environment and Climate Change Canada Ottawa Ontario Canada

**Keywords:** benthic, food web, isotopic niche, overlap, pelagic, stable isotope, sulfur

## Abstract

Each species occupies a distinct ecological niche, defined by a specific set of environmental conditions and resource requirements necessary for its survival and reproduction. However, climate change is altering species distributions, predator–prey relationships, and resource partitioning between species, with these changes being pronounced in the Arctic. Stable isotope analysis of carbon (δ^13^C) and nitrogen (δ^15^N) has been widely used to estimate isotopic niches and quantify niche overlap among species using a two‐dimensional approach (2D). However, δ^13^C is not always sufficient to differentiate habitat and resource use among species due to minimal variation between end‐members. Incorporating sulfur stable isotopes (δ^34^S) can enhance resolution in such cases. Using an Arctic coastal food web as a model system, we used a three‐dimensional isotopic niche approach (3D: δ^13^C–δ^15^N–δ^34^S) with 664 individuals across 49 species spanning multiple taxonomic groups (invertebrates, fish, seabirds, and marine mammals) that utilize resources from benthic and pelagic habitats. We compared the traditional 2D isotopic niches with a 3D framework using nicheROVER to assess how the addition of a third dimension changes niche size estimates and probability of niche overlap between species. We found that several benthic‐associated species, including the common eider ( *Somateria mollissima* ) and various benthic invertebrates, tended to show greater changes in isotopic niche size with the addition of δ^34^S than pelagic‐associated species. In addition, niche overlap among benthic‐associated taxa generally decreased with the 3D approach, suggesting better resolution of habitat use and resource partitioning. This reflects the greater ecological diversity, foraging specialization, and more complex food web structure characteristic of benthic than pelagic ecosystems. We recommend incorporating δ^34^S for aquatic studies that involve benthic habitats and emphasize the value of multidimensional approaches to increase the resolution of ecological niche analysis.

## Graphical abstract

We compared the traditional two‐dimensional (2D: δ^13^C and δ^15^N) isotopic niches with a three‐dimensional (3D: δ^13^C, δ^15^N, and δ^34^S) framework to evaluate changes in niche size and overlap. Overall, invertebrates and benthic‐associated species showed greater changes in niche size and reduced overlap using the 3D approach. These findings highlight the value of incorporating δ^34^S in food web studies, particularly for benthic ecosystems, and emphasize the importance of multidimensional approaches in ecological niche analysis.

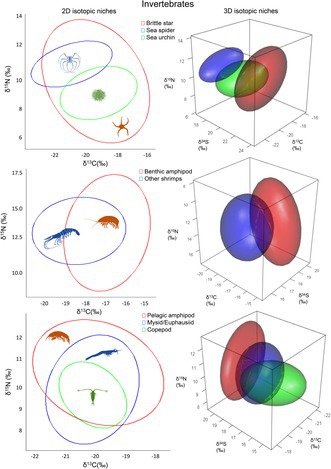

## 1 Introduction

Food webs are complex and dynamic, and understanding the interactions among species is essential for interpreting ecosystem structure and function. Different species can occupy similar feeding guilds and ecological roles (Blondel 2003), and determining the extent of their niche overlap is important for assessing the level of competition, resource partitioning, and trophic relationships in an ecosystem. Niche theory provides a foundational framework in ecology, describing how each species requires a specific set of environmental conditions and resources to survive and reproduce (Hutchinson 1957). This concept was expanded by Hutchinson (1957), who introduced the notion that the niche is an n‐dimensional hypervolume, where each axis represents a different environmental or resource variable necessary for the species to exist. These axes can be categorized as either bionomic—defined by biological resources and interspecific interactions (e.g., competition, predation)—or scenopoetic, which encompasses abiotic factors (e.g., temperature and salinity) (Hutchinson 1978). Within this framework, the fundamental niche encompasses the full range of conditions a species can tolerate in the absence of interspecific interactions such as competition and predation. In contrast, the realized niche reflects the actual conditions under which the species exists, constrained by interspecific interactions with other species that occupy similar habitat and utilize similar resources (Hutchinson 1957). As a result, the realized niche is typically narrower than the fundamental niche (Soberon and Arroyo‐Pena 2017).

In ecological studies, stable isotope analysis, particularly of carbon (δ^13^C) and nitrogen (δ^15^N), is widely used to estimate isotopic niches (Newsome et al. 2007; Jackson et al. 2011), identify food sources (Post 2002; Brown et al. 2016; Szpak and Buckley 2020; Cui et al. 2023), determine trophic position (Post 2002), assess spatial and temporal niche variability (Yurkowski et al. 2016), and quantify niche overlap among species (Jackson et al. 2011; Swanson et al. 2015). Stable isotope bi‐plots (i.e., δ^13^C and δ^15^N) are mainly used by ecologists to describe the isotopic niche; however the definition of Hutchison's niche as “n‐dimensional hypervolumes” implies niche as a multidimensional set of resources and therefore could include other stable isotopes or biological variables that would provide more resolution for estimating a species' niche and niche overlap between species (van Oordt et al. 2024).

The use of sulfur isotopes (δ^34^S) in food web studies has grown in recent years (Raoult et al. 2024). While δ^15^N is commonly used to infer trophic level, due to increases in δ^15^N of consumers up the food web (Hobson and Welch 1992; Post 2002), δ^13^C and δ^34^S are more indicative of resource use, as they generally exhibit minimal trophic fractionation (typically < 1‰). Carbon stable isotope ratios (δ^13^C) are often used to trace carbon sources in the food web. For instance, in a marine system, benthic primary producers (e.g., benthic algae and seagrass) generally have higher δ^13^C values than the pelagic producers like phytoplankton, allowing researchers to distinguish between benthic and pelagic food sources (Hobson and Welch 1992). In Arctic marine ecosystems, carbon sources such as ice algae and phytoplankton also differ in δ^13^C, with ice algae typically enriched in ^13^C (Hobson and Welch 1992; Hobson et al. 2002). Similarly, δ^34^S can differentiate pelagic and benthic food sources in the marine environment. In the water column, sulfur is primarily present as sulfate, while in the sediments, sulfur is often available as sulfides due to the activity of anaerobic bacteria present in the sediments (Peterson and Fry 1987; Peterson 1999). Sulfates and sulfides have distinct δ^34^S values and can therefore be used to distinguish between benthic and pelagic originating food sources, with the former being relatively lower in δ^34^S (Peterson and Fry 1987; Connolly et al. 2004). This isotopic distinction enables the identification of species that utilize resources from pelagic and benthic compartments, thereby revealing benthic–pelagic coupling in aquatic environments. Additionally, δ^34^S can be used to distinguish between freshwater and marine residency within estuarine systems, as δ^34^S values often follow a salinity gradient, with marine typically showing higher values compared to freshwater systems (Hesslein et al. 1991; Fry and Chumchal 2011). The application of δ^34^S has helped clarify habitat use and resource utilization across a wide range of taxa, including marine mammals (Szpak and Buckley 2020; Cani et al. 2024), sea turtles (Weber et al. 2023), sharks (Burke et al. 2024; Besnard et al. 2025), fish (Fry and Chumchal 2011; Cybulski et al. 2022), and invertebrates (Yamanaka et al. 2000; Bopp et al. 2023). In many cases, δ^34^S has revealed dietary patterns that δ^13^C alone could not detect, highlighting its value in a multi‐isotope approach.

Understanding food web and isotopic niche dynamics in the Arctic is especially critical, as the region is undergoing rapid environmental change, including ocean warming and extended periods of open water (Crawford et al. 2021) that has influenced species distributions and interspecific interactions (Dupont et al. 2024; Stroeve et al. 2024). These shifts have facilitated the northward expansion of sub‐Arctic species, potentially increasing interspecific competition and altering trophic relationships and niche dynamics within Arctic food webs (Fossheim et al. 2015; Dupont et al. 2024). Our comprehensive database includes multiple species of marine taxa from the Arctic, including 50 invertebrates, 14 fish, 2 seabirds, and 3 marine mammals species, all of which forage in either pelagic or benthic habitats. The main objective of this study is to demonstrate how the inclusion of δ^34^S alongside δ^13^C and δ^15^N can affect the ecological interpretation of isotopic niche analyses and to determine whether the magnitude of change varies across taxa and habitats for pelagic and benthic‐associated species. Specifically, we compared the traditional two‐dimensional (2D: δ^13^C–δ^15^N) approach to a three‐dimensional (3D: δ^13^C–δ^15^N–δ^34^S) framework to examine how isotopic niche overlap shifts with the addition of δ^34^S, and whether these changes vary between foraging habitats (pelagic and benthic) or among taxonomic groups: invertebrates, fish, seabirds, and marine mammals. We hypothesized that estimates of isotopic niche size would differ between 2D (δ^13^C–δ^15^N) and 3D (δ^13^C–δ^15^N–δ^34^S) approaches, and that these differences would vary among taxa and foraging habitats. Specifically, we expected isotopic niche size to be larger under the 3D framework for benthic species, reflecting the greater diversity of end‐member resources available in benthic habitats. We also predicted that isotopic niche overlap among taxa would decrease when using the 3D approach, particularly among benthic foragers, as the incorporation of δ^34^S should enhance discrimination among food resources used by each taxa.

## 2 Methods

### 2.1 Sample Collection

Samples of marine mammals, seabirds, fish, and invertebrates (*n* = 664, 49 species; Table S1) were collected around Southampton Island, Nunavut, Canada (64.5999° N, 84.1348° W) during the summer (June to September) over 3 years (2016, 2018, and 2019). In 2016, benthic and pelagic invertebrates and fish samples were collected using a Campelen 1200 trawl aboard the MV Nuliajuk. In 2018 and 2019, invertebrate and fish samples were collected aboard the RV William Kennedy using benthic beam trawls, Ponar grab and box core (25 × 25 × 50 cm) as part of the Southampton Island Marine Ecosystem Project (SIMEP). A 3‐m beam trawl (0.5‐cm cod‐end mesh) was towed (2–3 knots for 15 min) at the bottom to collect benthic fish and invertebrates. Zooplankton were collected with an obliquely towed bongo net (500‐μm mesh). All samples were sorted in the field to the lowest taxonomic group and frozen at −20°C for further analysis. The samples had their taxonomic identification visually verified in the lab to the lowest taxonomic group possible (i.e., class, genus or species) using taxonomic keys and guides for Arctic fish (Coad and Reist 2018) and invertebrates (Klekowski and Węsławski 1990; Lacasse et al. 2020). A total of 427 invertebrates and 133 fish, consisting of 32 and 12 species/taxa, respectively, were subsampled for further analysis.

Marine mammal muscle samples from beluga whale ( *Delphinapterus leucas* , *n* = 8), narwhal ( *Monodon monoceros* , *n* = 10), and ringed seal ( *Pusa hispida* , *n* = 40) subsistence harvest were collected by Naujaat (Arviq) community members during summer (2016, 2018, and 2019). Frozen tissues were shipped to the Freshwater Institute (Winnipeg, MB) as part of an ongoing community‐based monitoring program with Fisheries and Oceans Canada (DFO). Seabird blood samples (*n* = 46) from common eider ( *Somateria mollissima* ) and thick‐billed murre ( *Uria lomvia* ) were collected (2018 and 2019) by the long‐term monitoring program in East Bay and Coats Islands (Environment and Climate Change Canada—ECCC and McGill University, respectively). Plasma was analyzed for the common eiders sampled during pre‐incubation (at arrival; in May), while red blood cells (RBCs) were analyzed for thick‐billed murres sampled during chick‐rearing (in July). Since plasma has a turnover rate of a few hours to days (Hobson and Clark 1993; Hahn et al. 2012; Barquete et al. 2013), we expected that plasma samples collected from common eiders would reflect their local breeding diet during pre‐incubation rather than their migratory diet as samples were collected in May. In contrast, RBCs samples collected from thick‐billed murres during chick‐rearing should reflect diet over several weeks, again representing local breeding diets as samples were collected in July. Blood samples were shipped frozen (−20°C) to the Freshwater Institute (Winnipeg, MB) for analysis.

### 2.2 Stable Isotope Analysis

Muscle from fish and marine mammals, blood from seabirds and the whole body, gonad, muscle or soft tissues from invertebrates (Table S1) were subsampled and analyzed for carbon, nitrogen and sulfur stable isotopes (δ^13^C, δ^15^N, and δ^34^S). Frozen samples were freeze‐dried for 48 h at −50°C and homogenized prior to analysis. As higher lipid content can affect δ^13^C (Post et al. 2007), lipids were extracted from the invertebrate tissues and fish and marine mammal muscles using a modified 2:1 chloroform: methanol method developed by Bligh and Dyer (1959). For δ^13^C and δ^15^N, 400–600 μg of tissue was weighed into tin capsules while for δ^34^S, 3000–6000 μg of tissue was weighed into tin capsules with an additional 300–500 μg of Vanadium Pentoxide. Stable isotope analysis was performed using a Delta V Advantage Mass spectrometer (Thermo Finnigan, San Jose, CA, USA) coupled to a Costech 4010 Elemental Combustion system (Costech, Valencia, CA, USA) and a ConFlo IV gas interface (as described in Amiraux, Mundy, et al. 2023) at the Chemical Tracers Laboratory, Great Lakes Institute for Environmental Research (GLIER) at the University of Windsor, Ontario. Stable isotope ratios are expressed in per mil (‰) in standard delta (δ) notation relative to the international standards Pee Dee Belemnite (Carbon), atmospheric N_2_ (Nitrogen), and Vienna Canon Diablo Triolite (Sulfur). Instrument accuracy ranged from 0.06‰ to 0.14‰ for δ^15^N (NIST8573, NIST8547, NIST8574); 0.01 to 0.09‰ for δ^13^C (NIST8542, NIST8573, NIST8574); and 0.25 to 0.30‰ for δ^34^S (NIST8555, NIST8529). Instrument precision was measured as the standard deviation of standard replicates and was ≤ 0.25‰ for δ^15^N, ≤ 0.13‰ for δ^13^C (NIST1577c, internal lab standard, tilapia muscle, USGS 40 and Urea) and ≤ 0.43‰ for δ^34^S (USGS 42, NIST 8555 and NIST 8529).

As inorganic carbon can increase δ^13^C and does not reflect dietary sources, species with inorganic carbon present as calcium carbonate (CaCO_3_) such as sea stars, sea spiders, and brittle stars had their δ^13^C corrected mathematically in this study. We calculated the correct δ^13^C using the linear regression (Equation 1) derived from Jacob et al. (2005). The carbonate proxy used in the study was 0.9 as the average for echinoderms from Kazanidis et al. (2019).

(1) $$\delta^{13}C_{\text{crude}}-\delta^{13}C_{\text{acid}}=0.181+4.08\times \text{carbonate proxy}$$

### 2.3 Data Analysis

Species across four taxonomic classifications (invertebrates, fishes, marine mammals, and seabirds) were grouped into 29 different taxa (Table 1, Table S1). To ensure a sufficient sample size to estimate the isotopic niche for some taxa, we combined closely related species with similar foraging strategies with each taxa containing an average of 22 individuals (range = 8–111). To validate these groupings that contained multiple species (i.e., decapod, other shrimps; sculpins), we conducted multivariate analysis (Permutational Multivariate Analysis of Variance [PERMANOVA] and Principal Component Analysis [PCA]; Figures S1–S14) to ensure that the combined species exhibited appropriate clustering within the isotopic niche space using the three stable isotopes values (δ^13^C, δ^15^N, and δ^34^S). Each taxa were further assigned as benthic (*n* = 19) or pelagic (*n* = 10) based on their habitat as defined in World Register of Marine Species (WoRMS; https://marinespecies.org) and/or FishBase (https://fishbase.net.br). The classification allowed us to explore the magnitude of change in niche size and niche overlap from δ^13^C and δ^15^N (two dimensions) to δ^13^C, δ^15^N, and δ^34^S (three dimensions) isotopic niche analysis among taxa that inhabit and primarily forage on benthic or pelagic‐derived resources. Median sample size was higher for pelagic taxa (21.5; IQR: 11.75–36.25) than for benthic taxa (14; IQR: 12–19); therefore, comparisons between habitats should be interpreted with caution.

**TABLE 1:** Carbon, nitrogen, and sulfur stable isotope ratios (mean ± SD ‰) and two‐dimensional (‰^2^) and three‐dimensional (‰^3^) isotopic niche size (median and scaled) for each taxa (*n* = sample size) by foraging habitat.

Foraging habitat	Taxa	*n*	δ^13^C	δ^15^N	δ ^34^S	2D isotopic niche		3D isotopic niche	
						Niche size	Scaled niche size	Niche size	Scaled niche size
Pelagic	Ringed seal	40	−18.26 ± 0.39	17.53 ± 1.27	17.40 ± 0.54	7.33	−0.33	18.07	−0.41
	Narwhal	10	−18.13 ± 0.32	15.57 ± 1.38	17.53 ± 0.77	4.21	−0.72	10.69	−0.58
	Beluga	8	−18.28 ± 0.10	16.13 ± 0.41	17.46 ± 0.55	0.66	−1.17	1.47	−0.80
	Thick‐billed murre	31	−20.20 ± 0.37	14.84 ± 0.50	17.27 ± 0.26	2.86	−0.89	3.08	−0.76
	Arctic cod	14	−19.60 ± 0.79	14.28 ± 0.56	17.66 ± 0.70	7.27	−0.34	21.06	−0.35
	Mysid/Euphausiid	59	−20.10 ± 0.62	9.80 ± 0.88	18.14 ± 0.89	9.95	0.00	37.08	0.02
	Hydrozoan	11	−20.55 ± 0.86	11.44 ± 0.63	20.56 ± 0.94	8.05	−0.24	28.47	−0.18
	Copepod	22	−20.08 ± 0.43	9.59 ± 0.51	16.84 ± 0.94	3.73	−0.78	14.89	−0.49
	Chaetognath	21	−19.79 ± 0.58	13.35 ± 0.41	17.05 ± 0.62	3.35	−0.83	8.26	−0.64
	Pelagic amphipod	38	−19.90 ± 0.85	10.49 ± 0.84	19.47 ± 0.46	12.86	0.37	23.66	−0.29
Benthic	Common eider	15	−16.99 ± 96	13.26 ± 1.61	14.68 ± 3.61	24.25	1.80	204.26	3.86
	Arctic shanny	20	−19.15 ± 0.38	15.80 ± 0.60	17.53 ± 0.72	4.06	−0.74	12.53	−0.54
	Fourline snakeblenny	9	−18.58 ± 0.51	16.08 ± 0.48	16.40 ± 1.10	4.18	−0.72	16.55	−0.45
	Slender eelblenny	13	−17.70 ± 1.08	13.85 ± 0.76	15.39 ± 0.89	6.14	−0.48	11.86	−0.56
	Banded gunnel	14	−19.58 ± 0.55	14.57 ± 0.89	18.67 ± 0.26	7.17	−0.35	7.47	−0.66
	*Gymnocanthus* sp. sculpin	17	−16.35 ± 0.30	14.02 ± 0.55	13.90 ± 0.43	2.62	−0.92	4.24	−0.73
	*Myoxocephalus* spp. sculpin	18	−19.17 ± 0.86	15.51 ± 1.30	18.14 ± 1.39	19.55	1.21	86.15	1.15
	*Triglops* spp. sculpin	28	−19.65 ± 0.77	14.54 ± 0.68	18.13 ± 0.75	9.32	−0.08	24.79	−0.26
	Snail	9	−17.58 ± 0.86	14.07 ± 0.83	17.80 ± 1.23	8.89	−0.13	32.53	−0.08
	Sea urchin	12	−19.15 ± 0.88	9.04 ± 0.60	18.74 ± 0.71	8.90	−0.13	24.11	−0.28
	Sea star	12	−20.84 ± 1.06^a^	17.24 ± 1.22	21.44 ± 1.35	20.95	1.38	103.96	1.56
	Sea spider	9	−20.92 ± 0.88^a^	11.10 ± 0.49	17.79 ± 0.46	6.53	−0.43	11.98	−0.55
	Brittle star	16	−19.34 ± 1.29^a^	9.79 ± 1.45	21.39 ± 1.07	31.33	2.69	109.50	1.68
	Sea cucumber	13	−16.53 ± 0.57	11.71 ± 0.46	17.64 ± 0.82	4.62	−0.67	13.88	−0.51
	Nudibranch	10	−20.95 ± 0.21	13.19 ± 0.42	19.61 ± 0.59	1.31	−1.09	3.38	−0.75
	Isopod	14	−18.07 ± 0.72	9.90 ± 0.61	20.02 ± 0.44	6.83	−0.39	12.37	−0.55
	*Argis dentata*	23	−15.22 ± 1.17	14.38 ± 0.78	12.41 ± 1.58	15.76	0.73	68.25	0.74
	Other shrimps	111	−18.30 ± 0.94	13.12 ± 1.04	17.37 ± 0.80	18.35	1.06	64.31	0.65
	Benthic amphipod	47	−16.89 ± 0.86	12.98 ± 1.77	18.87 ± 0.54	27.50	2.21	69.22	0.76

^a^ Samples with δ^13^C mathematically corrected.

We used the nicheROVER package (Swanson et al. 2015) in R (4.3.1) to calculate the 95% niche size estimate of stable isotopes and the probability of niche overlap for each pairwise species combination (see Swanson et al. 2015) using two (2D_niche_: δ^13^C and δ^15^N) and three dimensions (3D_niche_: δ^13^C, δ^15^N, and δ^34^S). We used a threshold of 60% or higher to identify biologically significant niche overlap between taxa (Matley et al. 2017; Cybulski et al. 2022; Raoult et al. 2024). Estimated isotopic niche sizes (mean), calculated as Bayesian Ellipse Area (2D ‰^2^) and Bayesian Ellipsoid Volume (3D ‰^3^), were standardized (mean‐centered and scaled) to account for differences in measurement units and produce standardized isotopic niche sizes. This standardization removes unit‐specific biases and enables direct comparison between 2D and 3D niche sizes within taxa (Cybulski et al. 2022). Standardization was applied to posterior means rather than full posterior distributions; therefore, while this approach facilitates comparison among niche metrics, associated Bayesian uncertainty may not be fully propagated through the analysis. To assess the effect of including a third isotope, we calculated the degree of change as the difference between the standardized 3D and 2D niche sizes. A non‐parametric Kruskal–Wallis test was used to analyze differences in isotopic niche sizes among taxa inhabiting benthic and pelagic habitats.

## 3 Results

The mean values across all species for δ^13^C ranged from −20.95‰ ± 0.21‰ (nudibranch) to −15.22‰ ± 1.17‰ ( *Argis dentata* ), for δ^15^N from 9.04‰ ± 0.88‰ (sea urchin) to 17.53‰ ± 1.27‰ (ringed seal), and for δ^34^S from 12.41‰ ± 1.58‰ ( *A. dentata* ) to 21.44‰ ± 1.35‰ (sea stars) (Table 1; Figures S15–S18). The range of mean stable isotopes was greater across species foraging in the benthic habitat (δ^13^C: 5.4‰; δ^15^N: 8.2‰, and δ^34^S: 9.0‰) compared to those in the pelagic habitat (δ^13^C: 2.4‰; δ^15^N: 7.9‰, and δ^34^S: 3.7‰), with δ^13^C and δ^34^S ranges 2.3 and 2.4 times greater in benthic‐associated relative to pelagic‐associated species.

**FIGURE 1:**
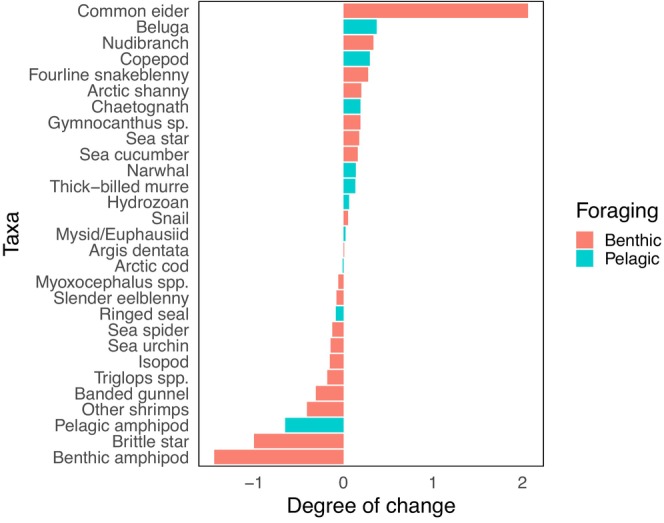
Degree of change between standardized isotopic niches (2D and 3D) for each taxa in descending order. Benthic taxa are shown in red and pelagic taxa in blue.

Isotopic niche size of species varied by foraging habitat and taxa. The 2D isotopic niche size (2D_niche_) in taxa that mainly forage in the benthic habitat was on average 12.01, ranging from 1.31 to 31.33‰^2^, compared to taxa that mainly forage in the pelagic habitat that was on average 6.03, ranging from 0.66 to 12.86‰^2^ (Table 1). The 3D isotopic niche size (3D_niche_) in benthic was on average 46.39, ranging from 3.38 to 204.26‰^3^ and for pelagic species the average was 16.67, ranging from 1.47 to 37.08‰^3^ (Table 2). Although the average isotopic niche size was higher in benthic than in the pelagic group for both 2D and 3D approaches, it was not statistically significant (2D: *χ* ^2^ = 2.43, *p* = 0.12; 3D: *χ* ^2^ = 2.02, *p* = 0.15). When comparing the 2D and 3D standardized isotopic niche sizes (2D_std.niche_ and 3D_std.niche_), the degree of change (niche difference) in benthic‐associated species was two times larger (mean degree of change = 0.39) than in pelagic‐associated species (mean degree of change = 0.20) (Figure 1). Common eider had the highest increase (2D_std.niche_ = 1.80; 3D_std.niche_ = 3.86) in isotopic niche size with the addition of δ^34^S, followed by beluga and nudibranch, while benthic amphipods had the largest decrease in isotopic niche size (2D_std.niche_ = 2.21; 3D_std.niche_ = 0.76), followed by brittle stars and pelagic amphipods (Figure 1). Overall, the isotopic niche overlap decreased when including δ^34^S for both pelagic and benthic habitats (Tables 2, 3, 4). For taxa in the benthic habitat, 37 out of 171 pairwise comparisons had biologically significant niche overlap (≥ 60%) when using only δ^13^C and δ^15^N (2D) and this level of overlap decreased to 14 of 171 with the addition of δ^34^S (3D). In the pelagic habitat, 10 out of 45 pairwise comparisons had biologically significant niche overlap when using the 2D approach, which decreased to 5 of 45 with δ^34^S (3D).

**TABLE 2:** Probability of isotopic niche overlap between taxa A (rows) with taxa B (columns) using 2D (δ^15^N and δ^13^C) and 3D (δ^15^N, δ^13^C, and δ^34^S) approaches in benthic invertebrates.

Benthic invertebrates		*Argis dentata*	Benthic amphipod	Brittle stars	Isopod	Nudibranch	Other shrimps	Sea cucumber	Sea spider	Sea star	Sea urchin	Snail
2D	*Argis dentata*		60	0	0	0	15	1	0	0	0	23
3D	*Argis dentata*		0	0	0	0	3	0	0	0	0	1
2D	Benthic amphipod	36		17	7	0	61	25	1	0	3	32
3D	Benthic amphipod	0		3	3	0	21	14	0	0	3	26
2D	Brittle stars	0	17		30	1	16	1	20	0	43	2
3D	Brittle stars	0	2		11	0	0	0	0	0	7	0
2D	Isopod	0	64	95		0	11	1	4	0	59	0
3D	Isopod	0	34	82		0	0	0	0	0	35	0
2D	Nudibranch	0	0	37	0		7	0	1	4	0	0
3D	Nudibranch	0	0	0	0		5	0	0	6	0	0
2D	Other shrimps	13	71	29	3	0		8	5	1	1	53
3D	Other shrimps	5	18	0	0	0		7	3	1	0	45
2D	Sea cucumber	2	98	15	1	0	50		0	0	0	1
3D	Sea cucumber	0	55	0	0	0	50		0	0	0	1
2D	Sea spider	0	2	89	6	0	13	0		0	2	2
3D	Sea spider	0	1	0	0	0	19	0		0	3	0
2D	Sea star	0	0	0	0	0	1	0	0		0	0
3D	Sea star	0	0	0	0	0	0	0	0		0	0
2D	Sea urchin	0	14	98	34	0	1	0	1	0		0
3D	Sea urchin	0	18	31	12	0	1	0	1	0		0
2D	Snail	50	91	8	0	0	86	1	1	0	0	
3D	Snail	4	48	0	0	0	65	1	0	0	0	

*Note:* Biologically significant overlaps (≥ 60%) are highlighted.

**FIGURE 2:**
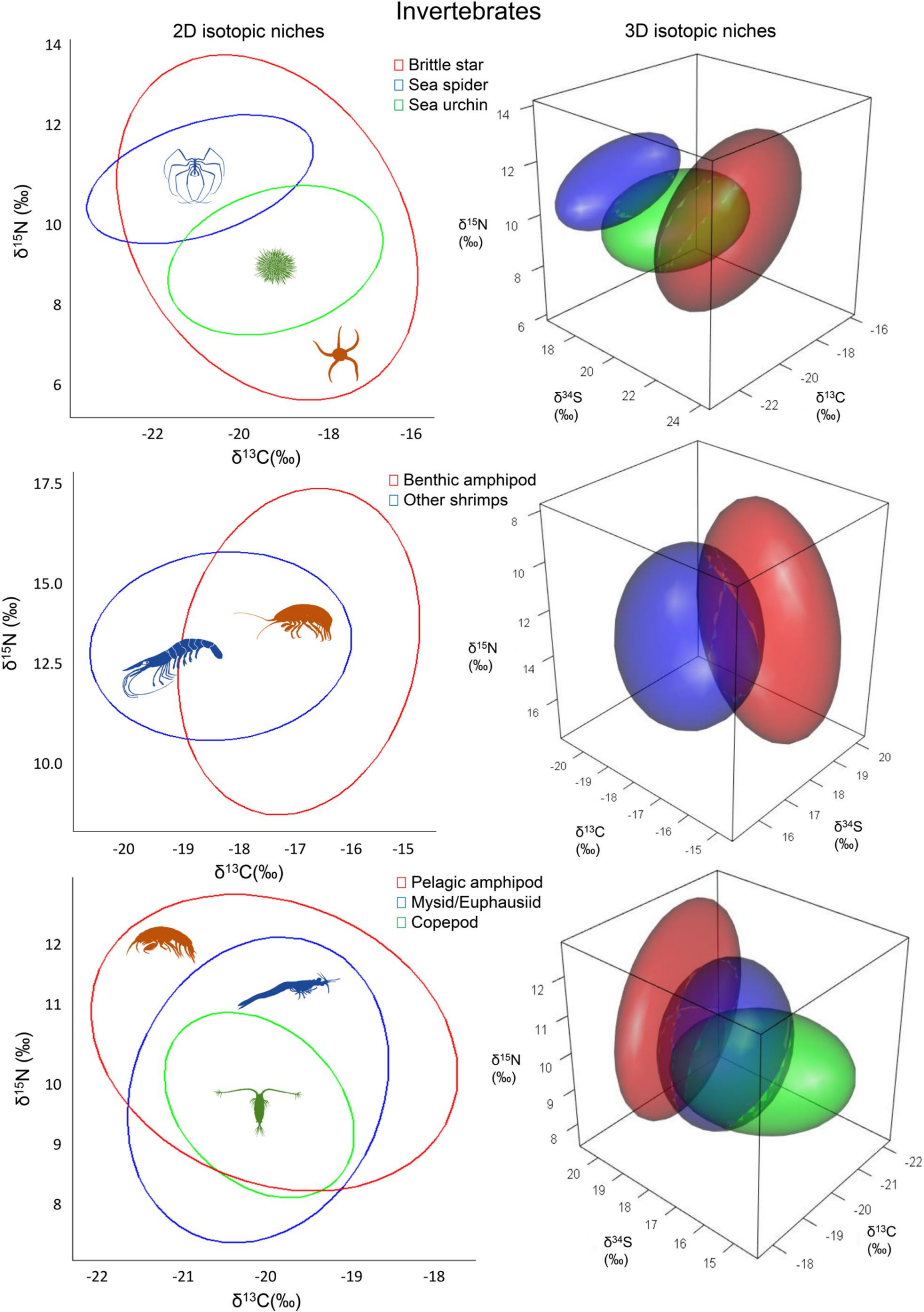
Two‐dimensional (2D: δ^13^C, δ^15^N) isotopic niche ellipses (left) and three‐dimensional (3D: δ^13^C, δ^15^N, δ^34^S) isotopic niche ellipsoids (right) representation of selected benthic and pelagic invertebrates that showed greater differences in niche overlap between the two approaches.

The probability of overlap of the common eider isotopic niche with other benthic species (benthic amphipods and other shrimps) decreased to below 25% when using the 3D approach with δ^34^S (2D: 88% and 61%). The benthic amphipod's standardized isotopic niche decreased with the 3D approach and had the second highest degree of change (Figure 1), decreasing the probability of isotopic niche overlap with *A. dentata* from 60% to 0%, other shrimps from 61% to 21%, and with common eider from 83% to 39% (Figure 2). Moreover, other shrimps, *Gymnocanthus* sp. sculpin, slender eelblenny, common eiders, isopods, and sea cucumbers, which previously had significant overlap (≥ 64%) with the benthic amphipods, had overlap percentages drop below 55% (Table 2). The standardized isotopic niche size of brittle stars also decreased with the addition of δ^34^S (Figure 1), reducing the probability of overlap with sea spider and sea urchins from ≥ 89% to ≤ 31% (Figure 2). Sea stars and nudribranch showed no significant overlap with any other benthic taxa using either the 2D or 3D approach (Table 2).

With the 3D isotopic niche approach, the pelagic amphipod reduced its standardized isotopic niche size and had the highest degree of change within the pelagic group (Figure 1), as reflected in the probability of overlap with other taxa. Species that previously fell within the pelagic amphipod's standardized isotopic niche, such as hydrozoans, mysids, and copepods, had their overlap percentages drop below 60% (2D: ≥ 83% to 3D: ≤ 42%; Table 3; Figure 2). Additionally, the pelagic amphipod's probability of falling into the mysid standardized isotopic niche decreased from 79% (2D) to 50% (3D), and the mysid's overlap with copepods decreased from 61% (2D) to 44% (3D) (Table 3).

**TABLE 3:** Probability of isotopic niche overlap between taxa A (rows) with taxa B (columns) using 2D (δ^15^N and δ^13^C) and 3D (δ^15^N, δ^13^C, and δ^34^S) approach in pelagic invertebrates.

Pelagic invertebrates		Chaetognath	Copepod	Hydrozoan	Mysid/Euphausiid	Pelagic amphipod
2D	Chaetognath		0	14	0	4
3D	Chaetognath		0	0	0	0
2D	Copepod	0		12	99	95
3D	Copepod	0		1	73	4
2D	Hydrozoan	4	6		46	83
3D	Hydrozoan	0	0		9	42
2D	Mysid/Euphausiid	0	61	25		87
3D	Mysid/Euphausiid	0	44	9		25
2D	Pelagic amphipod	1	36	45	79	
3D	Pelagic amphipod	0	6	46	50	

*Note:* Biologically significant overlaps (≥ 60%) are highlighted.

**FIGURE 3:**
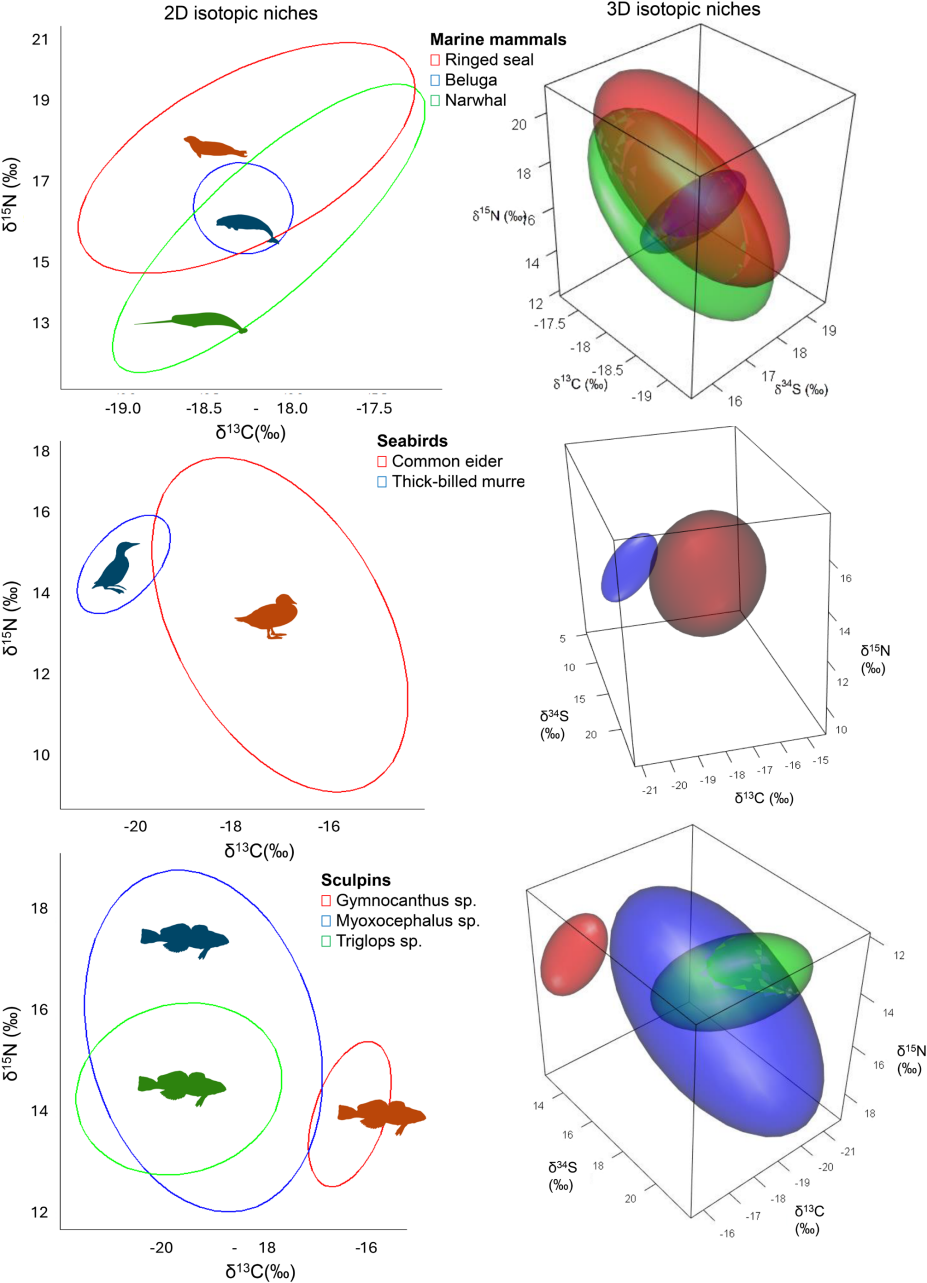
Two‐dimensional (2D: δ^13^C, δ^15^N) isotopic niche ellipses (left) and three‐dimensional (3D: δ^13^C, δ^15^N, δ^34^S) isotopic niche ellipsoids (right) representation of marine mammals (top), seabirds (center), and benthic fish (bottom) showing minimal change in niche overlap when using either 2D or 3D framework.

For fish, the addition of δ^34^S resulted in minimal changes to the standardized isotopic niche of Arctic cod (the only pelagic species) and its overall niche overlap with the benthic species. Using the 2D approach, Arctic cod showed significant isotopic niche overlap with two sculpins (*Myoxocephalu*s spp. 86% and *Triglops* spp. 92%) and banded gunnel (71%). When δ^34^S was incorporated (3D), the overlap probabilities decreased but generally remained significant (> 76%), with the exception of banded gunnel for which overlap decreased substantially to 15% (Table 4). Banded gunnel showed a significant isotopic niche overlap with Arctic cod, as well as with *Myoxocephalus* spp. and *Triglops* spp. sculpins, and these overlaps remained significant using either 2D (72%, 94%, 86%, respectively; Table 4) or 3D approaches (60%, 87%, 84%, respectively; Table 4). In addition, blennies (Arctic shanny and Fourline snakeblenny) and sculpins (*Myoxocephalus* spp. and *Triglops* spp.) showed significant isotopic niche overlap when using both the 2D and 3D approaches (64%–100%; Table 4). The isotopic niche overlap between the 3 sculpin species remained the same regardless of the use of 2D or 3D approach, with only *Triglops* spp. overlapping significantly with *Myoxocephalus* spp. (≥ 87%; Figure 3; Table 4). *Gymnocanthus* sp. and slender eelblenny had non‐significant overlap (Table 4) with other fish species regardless of the approach used. Overall, the addition of δ^34^S improved isotopic niche discrimination among only a few benthic fishes, reducing pairwise niche overlap estimates for Fourline snakeblenny–Arctic shanny and Arctic shanny–Fourline snakeblenny (65%–69% reduced to 44%–49%), as well as for *Triglops* spp.–banded gunnel (74% reduced to 31%; Table 4).

**TABLE 4:** Probability of isotopic niche overlap between taxa A (rows) using 2D (δ^15^N and δ^13^C) and 3D (δ^15^N, δ^13^C, and δ^34^S) approach in fish.

Fish		Arctic cod	Arctic shanny	Fourline snakeblenny	Slender eelblenny	Banded gunnel	*Gymnocanthus* sp.	*Myoxocephalus* spp.	*Triglops* spp.
2D	Arctic cod		22	7	6	71	0	86	92
3D	Arctic cod		23	2	3	15	0	88	76
2D	Arctic shanny	34		69	0	45	0	100	67
3D	Arctic shanny	31		49	0	11	0	97	64
2D	Fourline snakeblenny	16	65		0	11	0	96	35
3D	Fourline snakeblenny	4	44		0	0	0	66	19
2D	Slender eelblenny	13	0	0		21	15	53	16
3D	Slender eelblenny	8	0	0		0	10	54	2
2D	Banded gunnel	72	30	8	11		0	94	86
3D	Banded gunnel	60	25	1	0		0	87	84
2D	*Gymnocanthus* sp.	1	0	0	42	0		4	0
3D	*Gymnocanthus* sp.	0	0	0	38	0		2	0
2D	*Myoxocephalus* spp.	39	43	32	8	44	0		54
3D	*Myoxocephalus* spp.	33	34	15	4	13	0		39
2D	*Triglops* spp.	81	30	12	5	74	0	89	
3D	*Triglops* spp.	73	31	6	1	31	0	87	

*Note:* Biologically significant overlap (≥ 60%) are highlighted.

For marine mammals, the 3D isotopic niche of belugas still had significant overlap with those of narwhals (2D: 79%, 3D: 68%) and ringed seals (2D: 97%, 3D: 92%) (Table 5; Figure 3). A similar pattern was observed with thick‐billed murres and Arctic cod, where murres continued to show significant overlap with Arctic cod's isotopic niche (2D: 76%, 3D: 71%). Interestingly, narwhals, ringed seals, and thick‐billed murres had minimal to zero probability to overlap with other species' isotopic niches, regardless of the approach used. Overall, the addition of δ^34^S affected the isotopic niche overlap among the benthic‐associated taxa and invertebrates, but had a minimal influence on isotopic niche overlap on marine mammals.

**TABLE 5:** Probability of isotopic niche overlap between taxa A (rows) with taxa B (columns) using 2D (δ^15^N and δ^13^C) and 3D (δ^15^N, δ^13^C, and δ^34^S) approaches in seabirds and marine mammals.

			Common eider	Thick‐billed murre
Seabirds	2D	Common eider		0
	3D	Common eider		0
	2D	Thick‐billed murre	5	
	3D	Thick‐billed murre	5	

			Beluga	Narwhal	Ringed seal
Marine mammals	2D	Beluga		79	97
	3D	Beluga		68	92
	2D	Narwhal	16		51
	3D	Narwhal	15		51
	2D	Ringed seal	11	24	
	3D	Ringed seal	10	19	

*Note:* Biologically significant overlaps (≥ 60%) are highlighted.

## 4 Discussion

This is the first study to examine changes to isotopic niche dynamics and potential ecological interpretation when incorporating δ^34^S alongside traditional δ^13^C and δ^15^N values across numerous species and taxa that inhabit pelagic and benthic Arctic marine ecosystem. While previous research focused on a more limited number of species when comparing the use of 2D and 3D approaches (e.g., Rossman et al. 2016; Skinner et al. 2019; Cybulski et al. 2022; Raoult et al. 2024), our study includes multiple taxonomic groups (invertebrates, fishes, birds and marine mammals) and focuses on comparisons between distinct habitats (pelagic vs. benthic). By directly comparing benthic and pelagic environments, we address a key gap in food web ecology and demonstrate the added value of incorporating δ^34^S, particularly for species or habitat types where its inclusion enhances the resolution of trophic interactions and ecological interpretation.

Our analysis revealed that the degree of change between 2D (δ^13^C–δ^15^N) and 3D (δ^13^C–δ^15^N–δ^34^S) isotopic niches was more pronounced in invertebrates and benthic‐associated foragers. This distinction likely reflects the greater ecological complexity and diversity of foraging strategies characteristic of benthic ecosystems, in contrast to the more linear structure of pelagic food webs. In the Arctic, sympagic–pelagic–benthic coupling plays a key role in supporting benthic ecosystems where carbon inputs from pelagic sources (e.g., phytoplankton, ice algae and detritus) are transferred to the benthos (Stasko et al. 2018; Niemi et al. 2024). In addition, benthic organisms can acquire sulfur either from the water column, where it is present as sulfate with a higher δ^34^S value, or from the sediment, where it exists as sulfides with a lower δ^34^S value (Peterson 1999; Yamanaka et al. 2000). In addition, Southampton Island's Ordovician oil shales are geologically distinctive and may represent a local sulfur source throughout the region (Zhang 2011). These shales may be composed of more isotopically light sulfur and could contribute to lower benthic δ^34^S values, raising the possibility that part of the benthic sulfur signal observed in some taxa may reflect geological inputs, that can be incorporated biologically into the food web.

Among benthic invertebrates, several taxa exhibit high trophic plasticity, employing diverse feeding strategies such as suspension feeding, surface and subsurface deposit feeding, and predation/scavenging (Legeżyńska et al. 2012; Volage et al. 2021). These varied strategies are reflected in a broader range of stable isotope values (Christianen et al. 2017; Stasko et al. 2018). In our study, amphipods, brittle stars, and other shrimps showed substantial contraction in standardized isotopic niche size with the addition of δ^34^S. Amphipods, which display both scavenging and predatory behaviors, typically occupy mid‐trophic levels (TP: 2–3) (Amiraux, Mundy, et al. 2023; Ziegler et al. 2023; Bridier et al. 2024). Brittle stars are opportunistic feeders capable of switching between suspension and deposit feeding depending on food availability (Volage et al. 2021). Other shrimps included species from the genera *Eualus, Lebbeus*, and *Spirontocaris*, which are known to feed on a mix of amphipods, foraminiferans, hydrozoans, including some pelagic copepods (Birkely and Gulliksen 2003). This trophic plasticity allows these taxa to exploit a range of food sources in response to fluctuations in resource availability (e.g., ice algae vs. phytoplankton), which is an important adaptation to the variable Arctic environment. Relying solely on a 2D approach (δ^13^C and δ^15^N) may mask niche partition and lead to misinterpretation of ecological data, as the inclusion of δ^34^S (3D) provided a better resolution, revealing finer‐scale resource partitioning among these benthic taxa. In contrast, sea stars showed minimal change in isotopic niche size. Some species, such as those in the Pterasteridae family, function as top predators (TP: 4–5) (Amiraux, Yurkowski, et al. 2023) which likely explains their limited overlap with lower‐trophic benthic invertebrates. As opportunistic scavengers, sea stars can utilize both benthic and pelagic carrion (Garrido et al. 2021; Amiraux, Yurkowski, et al. 2023), which may explain the limited influence of δ^34^S on their isotopic niche size. Among seabirds, the inclusion of δ^34^S was more informative for common eiders, whose diet mainly consists of benthic invertebrates such as bivalves, polychaetas, amphipods, decapods, and echinoderms (Merkel et al. 2007; Kristjánsson et al. 2013). When δ^34^S was included, the isotopic niche of common eiders showed the most pronounced increase in size and decrease in overlap with other taxa, likely reflecting their specialized benthic foraging strategy focused on invertebrates. As observed in our study, species associated with benthic environments tend to show greater shifts in isotopic niche size and overlap when δ^34^S is incorporated in the analysis, highlighting the importance of the use of sulfur isotopes in ecological studies focused on species that rely primarily on benthic ecosystems. In contrast, thick‐billed murres are primarily pelagic feeders, consuming forage fish such as capelin ( *Mallotus villosus* ), Arctic cod and sandlance (*Ammodytes* spp.), as well as lower trophic‐level prey (i.e., copepods, amphipods, mysids) (Gaston and Noble 1985; Moody et al. 2012; Smith and Gaston 2012; Gaston and Elliott 2014; Góngora et al. 2018). Similar to other pelagic species, their standardized isotopic niche size showed minimal change with the addition of δ^34^S. Notably, thick‐billed murres had significant isotopic niche overlap with Arctic cod (with both 2D and 3D analyses), likely due to both species feeding on similar pelagic invertebrates such as copepods, amphipods and mysids in the area (Walkusz et al. 2013). In contrast to adults where invertebrates are a large part of the diet, murre chicks are fed primarily fish (Gaston and Noble 1985; Gaston and Elliott 2014).

As reported in a previous study (Fuirst et al. 2024), all fish species occupied similar trophic positions, ranging between 3 and 4. Standardized isotopic niche sizes among benthic fishes varied with the inclusion of δ^34^S where Arctic shanny, *Gymnocanthus* sp. and fourline snakeblenny showed an expansion in niche size under the 3D approach, whereas *Triglops* spp. and banded gunnel exhibited a reduction. Overall, the incorporation of δ^34^S did not substantially change or reduce isotopic niche overlap among benthic fish, except for Arctic shanny‐Fourline snakeblenny and Banded gunnel‐*Triglops* spp. sculpin pairs. *Gymnocamthus* sp. sculpin and slender eelblenny exhibited zero or low niche overlap with other benthic species when evaluated using either 2D or 3D isotopic approaches. Although these species were reported to have similar diets in the Siberian Arctic, feeding primarily on benthic amphipods and polychaetes (Gray et al. 2017), their isotopic niche overlap in present study was low and below significance (≤ 42%). In contrast, *Triglops* and *Myoxocephalus* sculpins are known to feed on a broader mix of benthic and bentho‐pelagic crustaceans (e.g., amphipods, mysids and shrimps), as well as other fishes (Gray et al. 2017; Landry et al. 2018; Tokranov et al. 2022), which likely explains the higher niche overlap with other benthic species and the more pelagic Arctic cod. Consistent with observations from the Siberian Arctic, *Gymnocamthus* sp. sculpin showed no overlap with the other two sculpin taxa.

In contrast, the standardized isotopic niche size of Arctic cod remained relatively unchanged with the addition of δ^34^S. Despite occupying a distinct pelagic habitat and feeding primarily on copepods, amphipods, and mysids (Walkusz et al. 2013; Majewski et al. 2016), Arctic cod still fell within the isotopic niche of 3 out of 7 benthic fish species in the 2D analysis. While generally considered pelagic, Arctic cod exhibit benthic–pelagic behavior where larger, older individuals (age + 1) tend to reside near the seafloor, feeding predominantly on amphipods, whereas smaller, younger fish (age 0) remain in the epipelagic zone, feeding mainly on copepods (Geoffroy et al. 2016; Majewski et al. 2016; Malizia et al. 2023). The individuals analyzed in the study were adults (+1) that can access deeper mesopelagic waters (Geoffroy et al. 2016), which likely contributed to the substantial isotopic niche overlap observed with benthic species such as *Myoxocephalus* spp. (≥ 86%) and *Triglops* spp. (≥ 76%) in both 2D and 3D isotopic space. Similar to Arctic cod, *M. scorpius* have also been reported to exhibit benthic–pelagic coupling, with larger individuals feeding more frequently on fish and smaller individuals relying primarily on benthic invertebrates (Landry et al. 2018). The inclusion of δ^34^S, however, reduced Arctic cod's overlap with banded gunnel, highlighting subtle differences in resource use between the two species. Specifically, banded gunnel fed on prey with higher δ^34^S values, suggesting greater reliance on pelagic sources compared to Arctic cod. Similarly, δ^34^S helped identify a higher proportion of pelagic inputs in sharks, highlighting its value in systems where benthic–pelagic coupling is expected (Raoult et al. 2024). These findings align with Cybulski et al. (2022), who demonstrated that δ^34^S can help distinguish isotopic niches among fish species that occupy different trophic guilds but share similar trophic positions.

For marine mammals, the small change in standardized isotopic niche size suggests limited value in incorporating δ^34^S to assess isotopic niche overlap among the three top marine predators. Regardless of whether a 2D or 3D isotopic space was used, belugas' isotopic niche fell within both narwhal's and ringed seal's isotopic niches, reflecting their shared reliance on pelagic prey. Beluga whales primarily consume pelagic fish, such as Arctic cod and capelin ( *M. villosus* ) (Loseto et al. 2009; Kelley et al. 2010; Choy et al. 2020). Similarly, narwhals predominantly feed on pelagic fish (e.g., Arctic cod), although some populations are known to feed on benthic fish, like Greenland halibut ( *Reinhardtius hippoglossoides* ) (Watt et al. 2013; Watt and Ferguson 2015). Ringed seals also primarily feed on pelagic fish (e.g., capelin, Arctic cod) and invertebrates (e.g., mysids, euphausiids, amphipods) (Yurkowski et al. 2016; Ogloff et al. 2019), but their diet is more variable, occasionally including benthic invertebrates (e.g., decapods) and fish (e.g., Stichaeidae) (Labansen et al. 2007; Schiøtt et al. 2024). Given the shared reliance on pelagic fishes, the δ^34^S values across these species were likely to overlap with minimal impact on the degree of change in isotopic niche size area (2D) and volume (3D). Similar to our study, the incorporation of δ^34^S values did not improved niche resolution among marine mammal species in coastal Africa (Cani et al. 2024). It is also important to note that these highly migratory predators may forage across multiple regions before feeding around Southampton Island, adding further uncertainty to the degree of isotopic separation that can be expected.

It is also important to note that the coupling between pelagic and benthic ecosystems is particularly tighter in the Arctic compared to warmer regions, mainly driven by its strong seasonality in primary production and the rapid sinking of ice algae to the seafloor (Darnis et al. 2012). Values of δ^34^S have been shown to be a valuable indicator to differentiate between benthic and pelagic feeding habitats in marine ecosystems (Szpak and Buckley 2020; Cybulski et al. 2022; Raoult et al. 2024). It has also been used to assess habitat use in estuaries (Connolly et al. 2004), freshwater lakes (Croisetiere et al. 2009; Onishi et al. 2023), and inshore‐offshore gradient (Rossman et al. 2016). The interpretation of δ^34^S values is often complicated by regional variability in benthic–pelagic coupling, particularly in the Arctic where the flux of sinking pelagic organic matter to the seafloor varies across regions (Stasko et al. 2018). In areas with stronger pelagic input, benthic species may exhibit elevated δ^34^S values due to the incorporation of pelagic‐derived sulfur. Conversely, some pelagic species may feed on resuspended benthic organic matter, resulting in lower δ^34^S values that can obscure their true foraging strategies. In other aquatic systems, such as estuaries, the gradient of salinity drives the δ^34^S values as the sulfate present in saltwater is typically higher in δ^34^S compared to freshwater (Connolly et al. 2004; Fry and Chumchal 2011). A similar pattern is observed along inshore–offshore gradients, where terrestrial inputs tend to lower δ^34^S values relative to more pelagic, offshore zones (Cani et al. 2023; Karalis et al. 2025). These complexities reinforce the importance of δ^34^S as a tool for investigating isotopic niche dynamics in aquatic ecosystems, while also emphasizing the need for careful consideration of regional and ecological context when interpreting niche size and overlap among species. Finally, niche estimates derived from some taxa with low sample sizes (< 10) should be interpreted with some caution, as mean isotopic niche estimates, and particularly the credible intervals around those estimates, are sensitive to sampling effort (Syväranta et al. 2013; Rossman et al. 2016).

## 5 Conclusion

This study is the first to investigate changes in isotopic niche dynamics and ecological interpretations by integrating δ^34^S with the more commonly used δ^13^C and δ^15^N across multiple taxonomic groups—including invertebrates, fishes, birds, and marine mammals—that utilize resources from benthic and pelagic environments. Our findings suggest that the niche of benthic‐associated species tended to be more influenced by the inclusion of δ^34^S, highlighting its potential to substantially improve resolution in food web studies focused on species that inhabit or rely on benthic environment. It is also important to acknowledge the practical limitations associated with δ^34^S analysis. Compared to δ^13^C and δ^15^N, δ^34^S typically requires larger sample sizes and incurs higher analytical costs, as it is analyzed separately. In some cases, these additional requirements may not be justified, particularly if δ^34^S does not substantially enhance ecological interpretations for certain species, taxa, or habitats. Nevertheless, incorporating δ^34^S remains valuable, especially when estimating isotopic niche overlap among species. This is particularly true for benthic‐associated and benthic‐pelagic coupling species, where δ^34^S can offer critical insights into resource partitioning and habitat use that may not be captured by carbon and nitrogen stable isotopes alone. In addition, the use of multiple stable isotopes (i.e., δ^34^S, δ^2^H, δ^18^O) can enhance trophic resolution in other complex ecosystems such as freshwater, estuarine, coastal, terrestrial environments, broadening the applicability of this approach beyond the Arctic marine environment.

## Author Contributions

**Paloma C. Carvalho:** conceptualization (equal), formal analysis (lead), investigation (equal), visualization (lead), writing – original draft (lead). **Kelsey F. Johnson:** conceptualization (equal), formal analysis (supporting), investigation (equal), writing – review and editing (equal). **Kyle H. Elliott:** data curation (equal), funding acquisition (equal), resources (equal), writing – review and editing (equal). **Steven H. Ferguson:** data curation (equal), funding acquisition (equal), resources (equal), writing – review and editing (equal). **Aaron T. Fisk:** data curation (equal), funding acquisition (equal), resources (equal), writing – review and editing (equal). **H. Grant Gilchrist:** data curation (equal), funding acquisition (equal), resources (equal). **Kevin J. Hedges:** data curation (equal), funding acquisition (equal), resources (equal). **Oliver P. Love:** data curation (equal), funding acquisition (equal), resources (equal). **C. J. Mundy:** data curation (equal), funding acquisition (equal), resources (equal), writing – review and editing (equal). **Andrea Niemi:** data curation (equal), funding acquisition (equal), resources (equal), writing – review and editing (equal). **Wesley R. Ogloff:** data curation (equal), resources (equal), writing – review and editing (equal). **Bruno Rosenberg:** data curation (equal), methodology (equal), resources (equal). **Cortney A. Watt:** data curation (equal), funding acquisition (equal), resources (equal), writing – review and editing (equal). **David J. Yurkowski:** conceptualization (equal), data curation (equal), formal analysis (supporting), funding acquisition (equal), project administration (lead), resources (equal), writing – original draft (supporting), writing – review and editing (lead).

## Funding

This study was supported by MEOPAR, NSERC, Churchill Marine Observatory (CFI), Arctic Research Foundation, and Fisheries and Oceans Canada.

## Conflicts of Interest

The authors declare no conflicts of interest.

## Supporting information


**Data S1:** ece373671‐sup‐0001‐DataS1.xlsx.

**Data S2:** ece373671‐sup‐0002‐Supinfo.docx.

## Data Availability

The data that supports the findings of this study are available in the Supporting Information of this article.
