# Supplementary material for: 2D to 3D: Exploring Variation of Niche Dimensionality Across Consumers in a Coastal Arctic Ecosystem and Implications on Interpretation
Source: Ecol Evol. 2026 May 13;16(5):e73671. doi: 10.1002/ece3.73671 (PMC13171226; doi:10.1002/ece3.73671)
Supplement: Supplementary file 2 — Data S2: ece373671‐sup‐0002‐Supinfo.docx. [file ECE3-16-e73671-s002.docx]

Table S1. List of species/taxa with their sample size (n) and type of tissue sampled for stable isotope analysis classified by foraging habitat.

| **Foraging habitat** | **Taxa** | **Tissue** | **Lowest taxonomic level** | **n** |
| --- | --- | --- | --- | --- |
| **Pelagic** | Ringed seal | Muscle | *Pusa hispida* | 40 |
|  | Narwhal | Muscle | *Monodon monoceros* | 10 |
|  | Beluga | Muscle | *Delphinapterus leucas* | 8 |
|  | Thick-billed murre | Blood | *Uria lomvia* | 31 |
|  | Arctic cod | Muscle | *Boreogadus saida* | 14 |
|  | Mysids/Euphausiids | Whole body | *Meganyctiphanes norvegica* | 3 |
|  |  |  | *Mysis oculata* | 17 |
|  |  |  | *Thysanoessa inermis* | 10 |
|  |  |  | *Thysanoessa raschii* | 29 |
|  | Hydrozoan | Whole body | Hydrozoa | 11 |
|  | Copepod | Whole body | *Calanus hyperboreus* | 20 |
|  |  |  | *Metridia sp.* | 2 |
|  | Chaetognath | Whole body | *Chaetognatha* | 21 |
|  | Pelagic amphipod | Whole body | *Themisto libellula* | 38 |
| **Benthic** | Common eider | Plasma | *Somateria mollissima* | 15 |
|  | Fourline snakeblenny | Muscle | *Eumesogrammus praecisus* | 9 |
|  | Arctic shanny | Muscle | *Stichaeus punctatus* | 20 |
|  | Slender eelblenny | Muscle | *Lumpenus fabricii* | 13 |
|  | Banded gunnel | Muscle | *Pholis fasciata* | 14 |
|  | Triglops spp. sculpin | Muscle | *Triglops sp.* | 3 |
|  |  |  | *Triglops pingelii* | 5 |
|  |  |  | *Triglops murrayi* | 20 |
|  | Myoxocephalus spp. sculpin | Muscle | *Myoxocephalus sp* | 2 |
|  |  |  | *Myoxocephalus scorpius* | 9 |
|  |  |  | *Myoxocephalus scorpioides* | 7 |
|  | Gymnocanthus sp. | Muscle | *Gymnocanthus tricuspis* | 17 |
|  | Snail | Soft parts | *Buccinum sp.* | 9 |
|  | Sea urchin | Gonads | *Strongylocentrotus droebachiensis* | 12 |
|  | Sea star | Whole body | *Crossaster papposus* | 1 |
|  |  |  | *Diplopteraster multipes* | 4 |
|  |  |  | *Henricia sp.* | 2 |
|  |  |  | *Pteraster militaris* | 3 |
|  |  |  | *Solaster sp.* | 2 |
|  | Sea spider | Whole body | Nymphonidae | 9 |
|  | Brittle star | Disk, arms | *Ophiopholis aculeata* | 16 |
|  | Sea cucumber | Muscle, Whole body | *Molpadia sp.* | 13 |
|  | Nudibranch | Whole body | *Dendronotus sp.* | 10 |
|  | Isopod | Whole body | *Arcturus baffini* | 14 |
|  | Argis dentata | Muscle | *Argis dentata* | 23 |
|  | Other shrimps | Muscle | *Eualus fabricii* | 10 |
|  |  |  | *Eualus gaimardii* | 28 |
|  |  |  | *Lebbeus groenlandicus* | 9 |
|  |  |  | *Lebbeus polaris* | 54 |
|  |  |  | *Spirontocaris spinus* | 10 |
|  | Benthic amphipod | Whole body | *Anonyx sp.* | 31 |
|  |  |  | *Eusirus cuspidatus* | 3 |
|  |  |  | *Haploops tubicula* | 1 |
|  |  |  | *Paramphithoe hystrix* | 1 |
|  |  |  | *Rhachotropis aculeata* | 11 |

**Preliminary analysis on taxa grouping using multivariate analysis: Permutational Multivariate Analysis of Variance (PERMANOVA) and Principal Component Analysis (PCA)**

**Benthic Amphipods**

PERMANOVA indicated that species had a significant effect on multivariate distances (F = 4.22, R² = 0.287, p = 0.004), explaining about 29% of the variation in the distance matrix. However, visual inspection of the PCA (Fig. S1) showed substantial overlap among species, and given the relatively low proportion of variance explained (<30%), all species were retained and analyzed together.


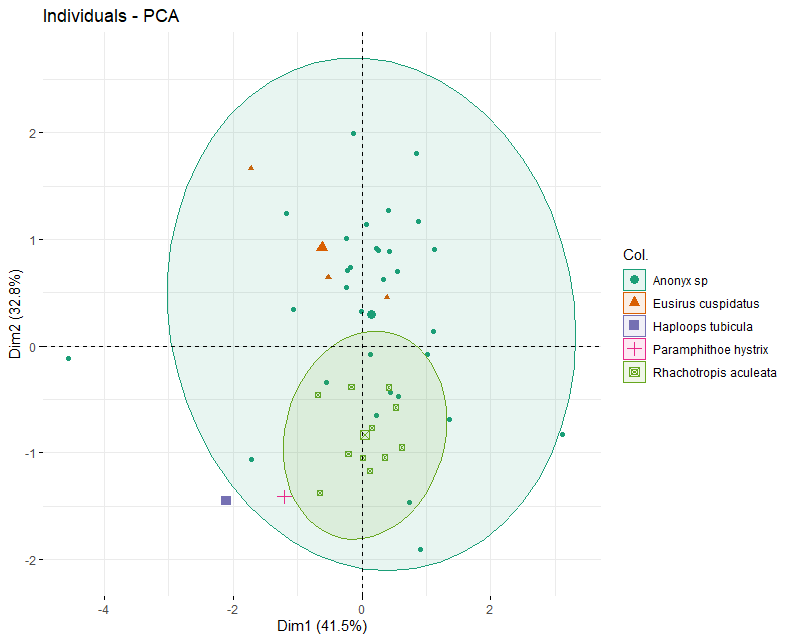


Figure S1. Principal component analysis (PCA) using δ¹³C, δ¹⁵N, and δ³⁴S stable isotope values for benthic amphipods.

**Blennies**

PERMANOVA showed a strong and significant effect of species on multivariate distances (F = 21.53, R² = 0.606, p = 0.001), indicating that species explained approximately 61% of the variation in the distance matrix. Based on PCA (Fig. S2), species were analyzed separately, and *Leptoclinus maculatus* was removed due to low sample size (n = 4).


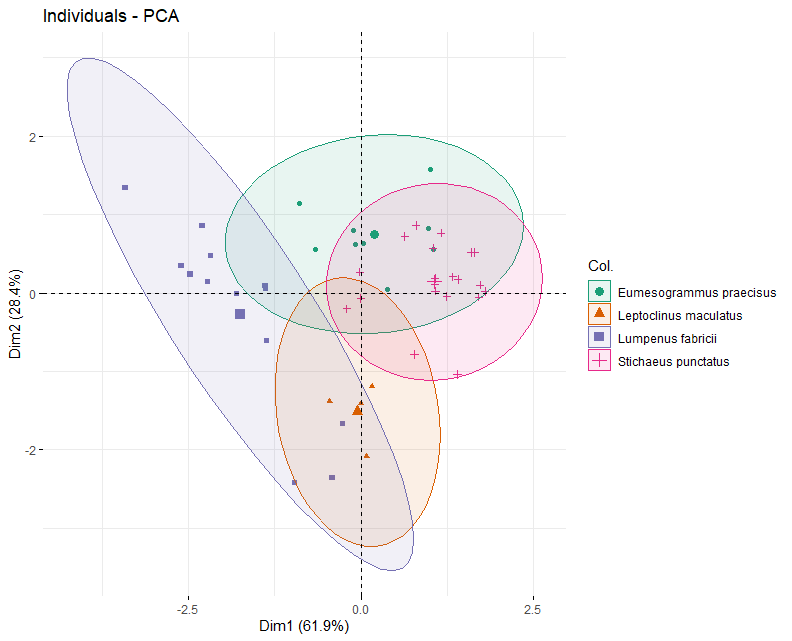


Figure S2. Principal component analysis (PCA) using δ¹³C, δ¹⁵N, and δ³⁴S stable isotope values for blennies.

**Copepods**

PERMANOVA indicated that species had a significant effect on multivariate distances (F = 4.48, R² = 0.183, p = 0.008), explaining approximately 18% of the variation in the distance matrix. However, based on PCA results (Fig. S3) and the relatively low proportion of variation explained (<20%), all species were kept together for the analysis.


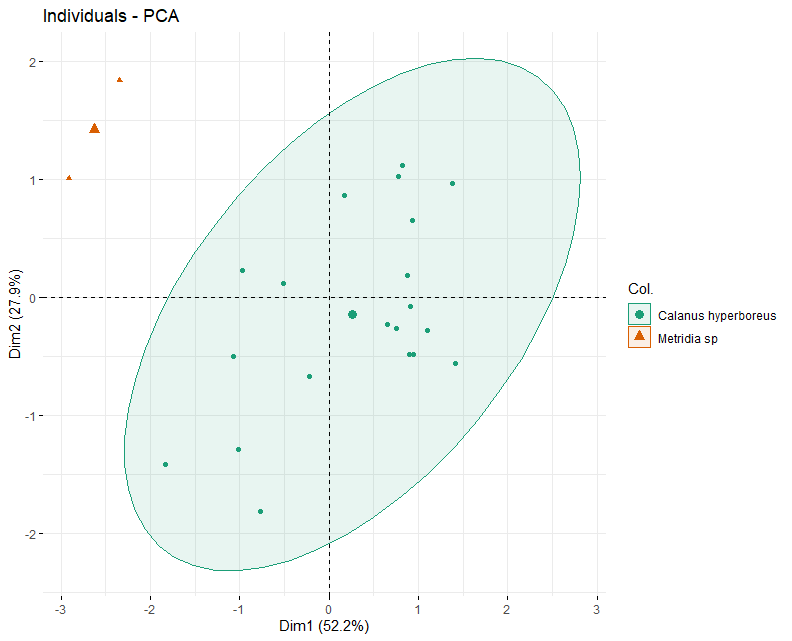


Figure S3. Principal component analysis (PCA) using δ¹³C, δ¹⁵N, and δ³⁴S stable isotope values for copepods.

**Decapods**

PERMANOVA revealed a strong and highly significant effect of species on multivariate distances (F = 35.69, R² = 0.712, p = 0.001), indicating that species explained approximately 71% of the total variation in the distance matrix. Based on PCA results (Fig. S4), *Argis dentata* was clearly separated from other shrimp species and was therefore analyzed separately. The remaining shrimp species were pooled as “Other shrimps” (Fig. S5), and species with low sample sizes (*Pagurus* sp. *Pandalus montagui, Sclerocragon boreas* and *Spirontocaris phippsi* n<3) were removed from the analysis.


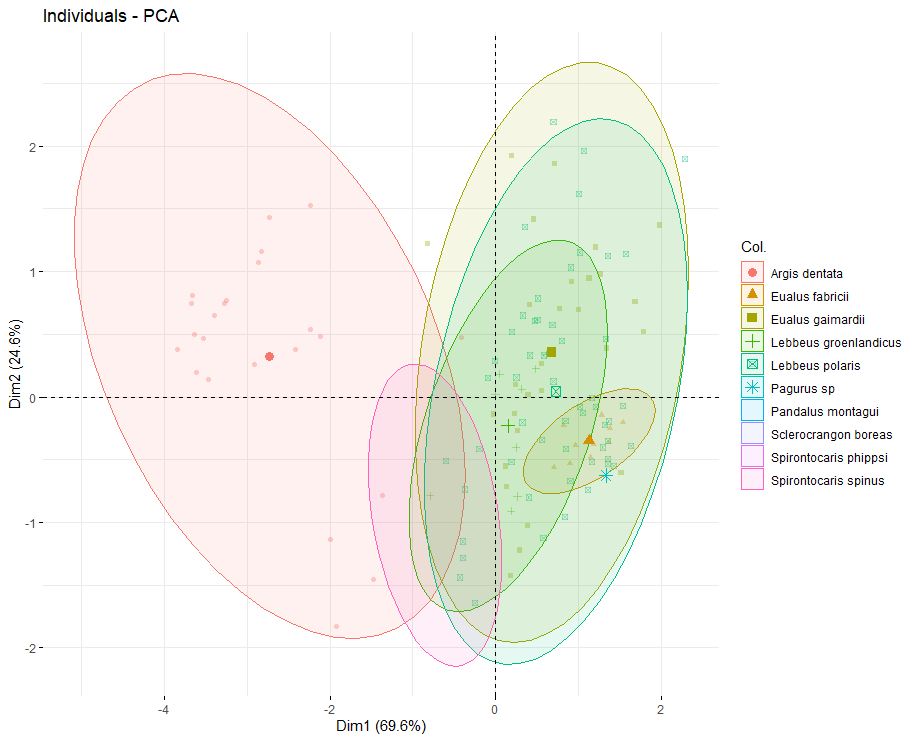


Figure S4. Principal component analysis (PCA) using δ¹³C, δ¹⁵N, and δ³⁴S stable isotope values for decapods.

**Decapod: Other shrimps**

PERMANOVA indicated significant differences among species (F = 9.30, R² = 0.260, p = 0.001), indicating that species explained approximately 26% of the total variation in the distance matrix. However, based on PCA results (Fig. S5) and the relatively low proportion of variation explained (<30%), all shrimp species were kept together for the analysis.


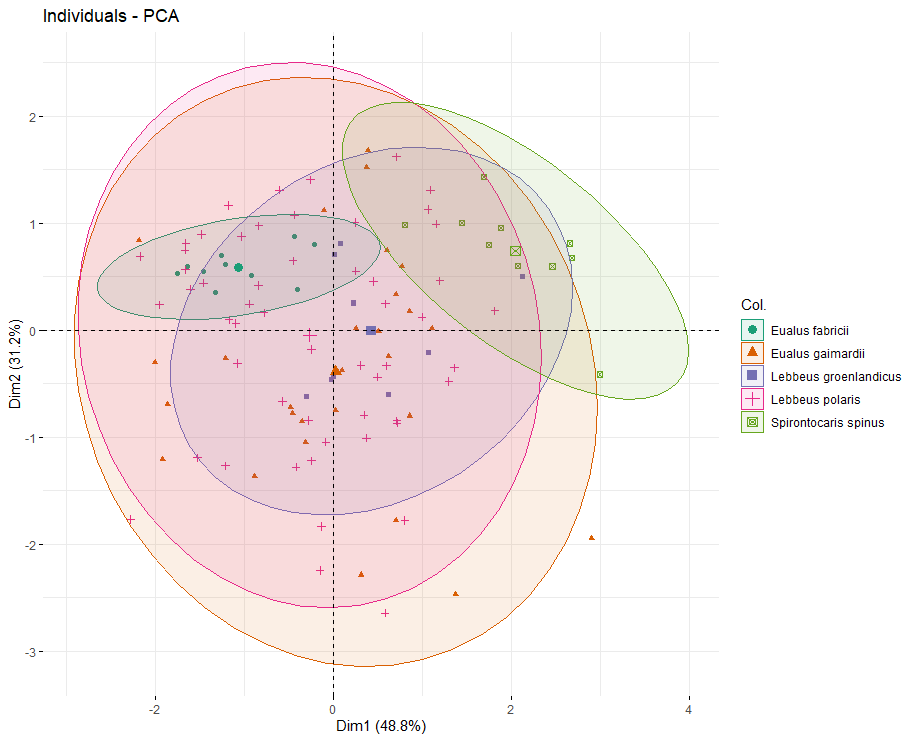


Figure S5. Principal component analysis (PCA) using δ¹³C, δ¹⁵N, and δ³⁴S stable isotope values for other shrimps.

**Mysids/Euphausiids**

PERMANOVA showed that species had a significant effect on multivariate distances (F = 6.53, R² = 0.263, p = 0.001), indicating that species explained approximately 26% of the variation in the distance matrix. However, based on PCA results (Fig. S6) and the relatively low proportion of variation explained (<30%), all species were kept together for the analysis.
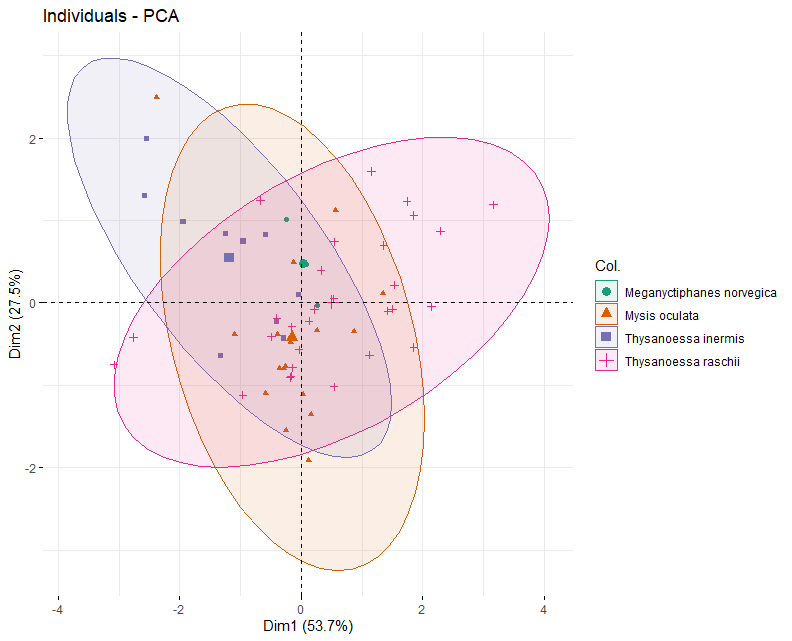


Figure S6. Principal component analysis (PCA) using δ¹³C, δ¹⁵N, and δ³⁴S stable isotope values for mysids/euphausiids.

**Pelagic Amphipod**

PERMANOVA showed that species had a strong and significant effect on multivariate distances (F = 20.39, R² = 0.327, p = 0.001), indicating that species explained approximately 33% of the variation in the distance matrix. Based on PCA results (Fig. S7), *Themisto abyssorum* was separated from the other *Themisto* and removed from subsequent analyses.
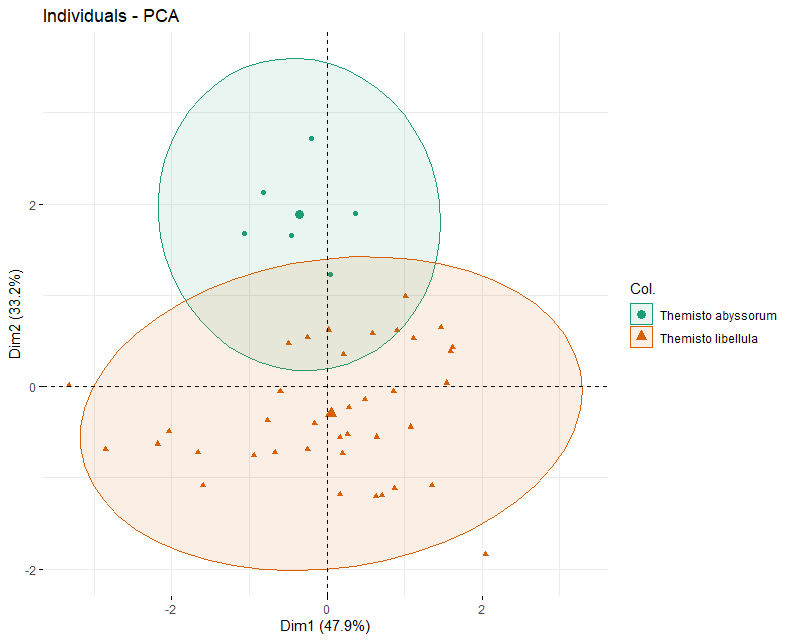


Figure S7. Principal component analysis (PCA) using δ¹³C, δ¹⁵N, and δ³⁴S stable isotope values for pelagic amphipods.

**Sculpins**

PERMANOVA showed a strong and highly significant effect of species on multivariate distances (F = 27.72, R² = 0.776, p = 0.001), indicating that species explained approximately 78% of the total variation in the distance matrix. Based on visual inspection of the PCA (Fig. S8), samples were grouped at the genus level. PCA supported this grouping (Fig. S9), resulting in three genera (*Gymnocanthus sp., Myoxocephalus spp., and Triglops spp*.). The genus with insufficient sample size (*Icelus*; n = 1) was removed from the analysis.


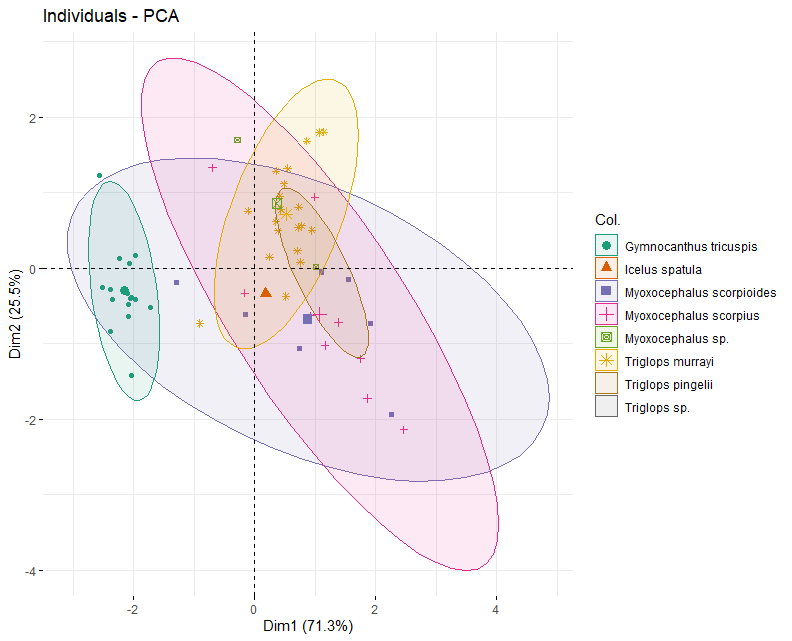


Figure S8. Principal component analysis (PCA) using δ¹³C, δ¹⁵N, and δ³⁴S stable isotope values for sculpins.

**
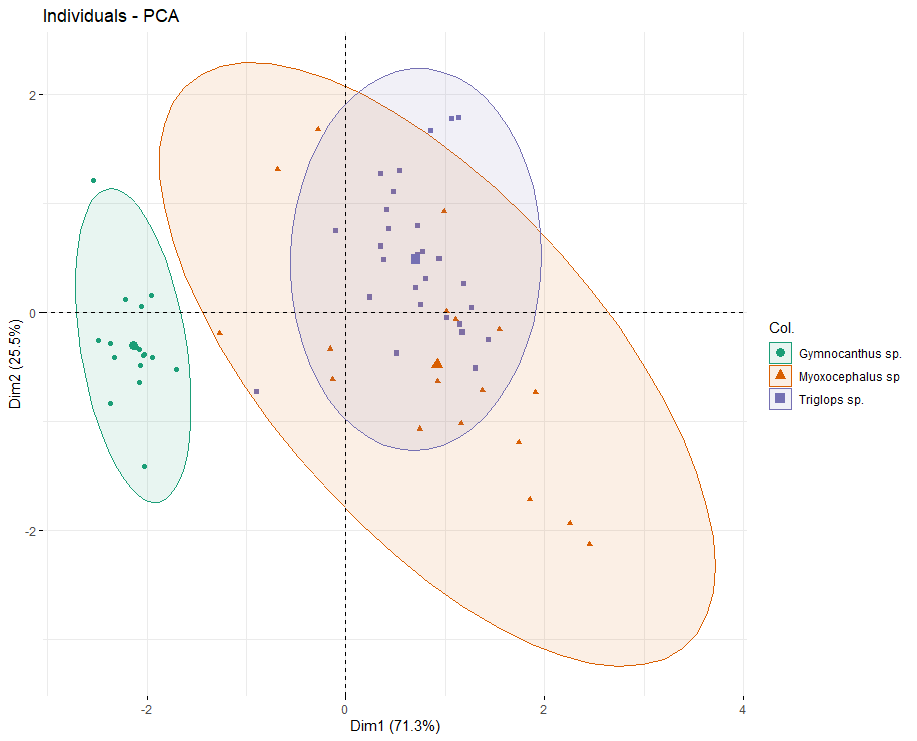
**

Figure S9. Principal component analysis (PCA) using δ¹³C, δ¹⁵N, and δ³⁴S stable isotope values for the 3 sculpins genera.

**Sea cucumbers**

PERMANOVA showed that species had a strong and significant effect on multivariate distances (F = 19.05, R² = 0.803, p = 0.001), indicating that species explained approximately 80% of the variation in the distance matrix. Based on PCA results (Fig. S10), only *Molpadia* sp. (n=13) was retained for subsequent analyses.


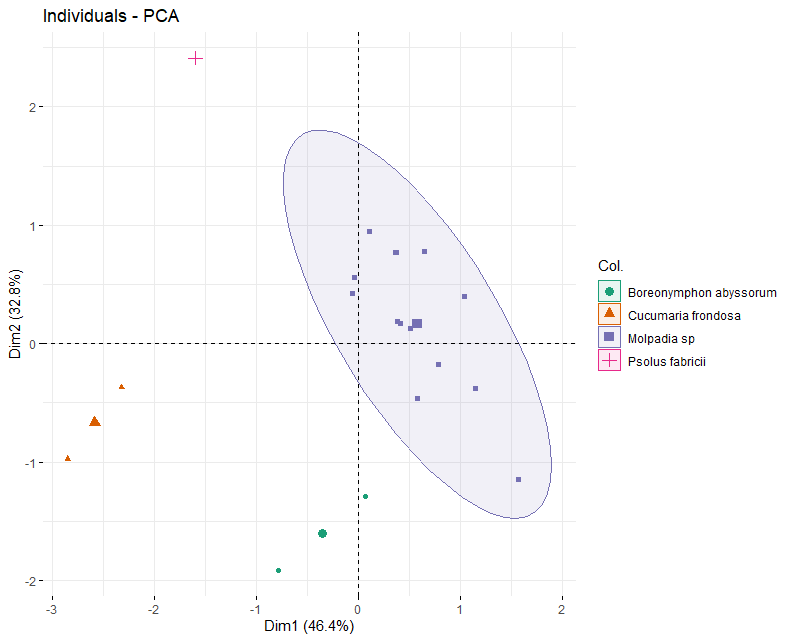


Figure S10. Principal component analysis (PCA) using δ¹³C, δ¹⁵N, and δ³⁴S stable isotope values for sea cucumbers.

**Sea stars**

PERMANOVA indicated that species did not have a significant effect on multivariate distances (F = 1.68, R² = 0.490, p = 0.143). Although species explained approximately 49% of the variation in the distance matrix, this effect was not statistically significant. Based on PCA results (Fig. S11), which showed substantial overlap among taxa, all species were kept together for the analysis.
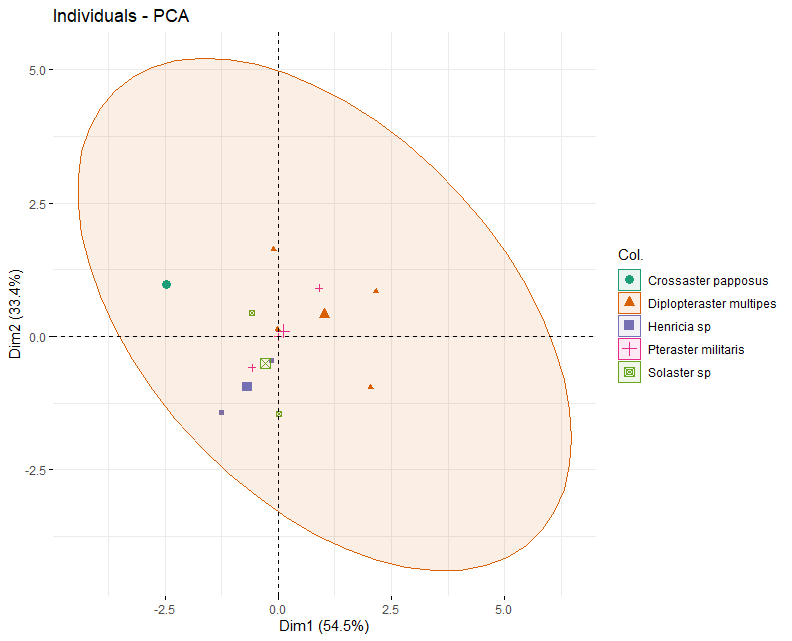


Figure S11. Principal component analysis (PCA) using δ¹³C, δ¹⁵N, and δ³⁴S stable isotope values for sea stars.

**Snail**

PERMANOVA showed that species had a strong and significant effect on multivariate distances (F = 8.75, R² = 0.686, p = 0.001), indicating that species explained approximately 69% of the variation in the distance matrix. Based on PCA results (Fig. S12), only *Buccinum* sp. (n=9) was retained for the analysis.


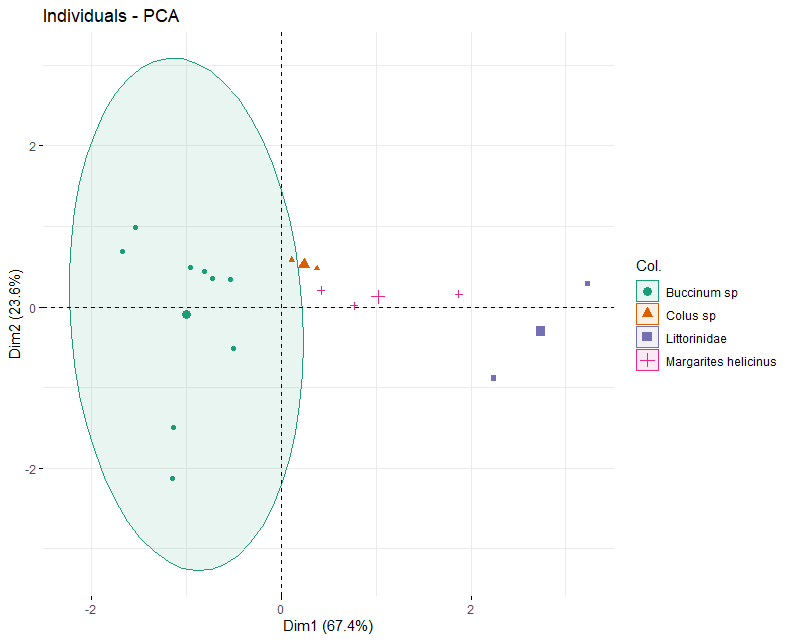


Figure S12. Principal component analysis (PCA) using δ¹³C, δ¹⁵N, and δ³⁴S stable isotope values for snails.

**Worms**

PERMANOVA showed that species had a significant effect on multivariate distances (F = 3.53, R² = 0.391, p = 0.015), indicating that species explained approximately 39% of the variation in the distance matrix. However, based on PCA results (Fig. S13) and the small sample size of individuals per species, this taxa was removed from the analysis.


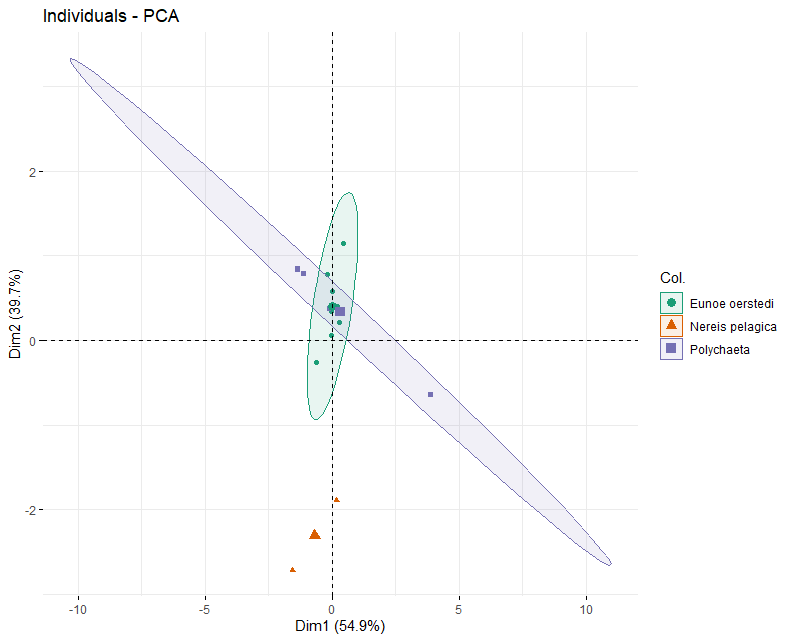


Figure S13. Principal component analysis (PCA) using δ¹³C, δ¹⁵N, and δ³⁴S stable isotope values for worms.

**Bivalves**

PERMANOVA showed that species had a significant effect on multivariate distances (F = 8.09, R² = 0.802, p = 0.009), indicating that species explained about 80% of the variation in the distance matrix. Based on PCA results (Fig. S14) and the small sample size per species, these taxa (bivalves) were removed from the analysis.


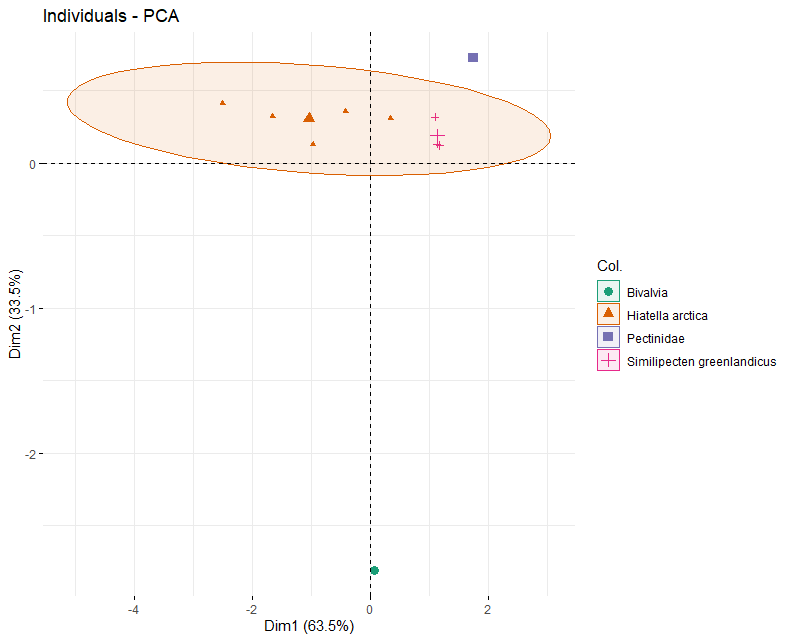


Figure S14. Principal component analysis (PCA) using δ¹³C, δ¹⁵N, and δ³⁴S stable isotope values for bivalves.

**
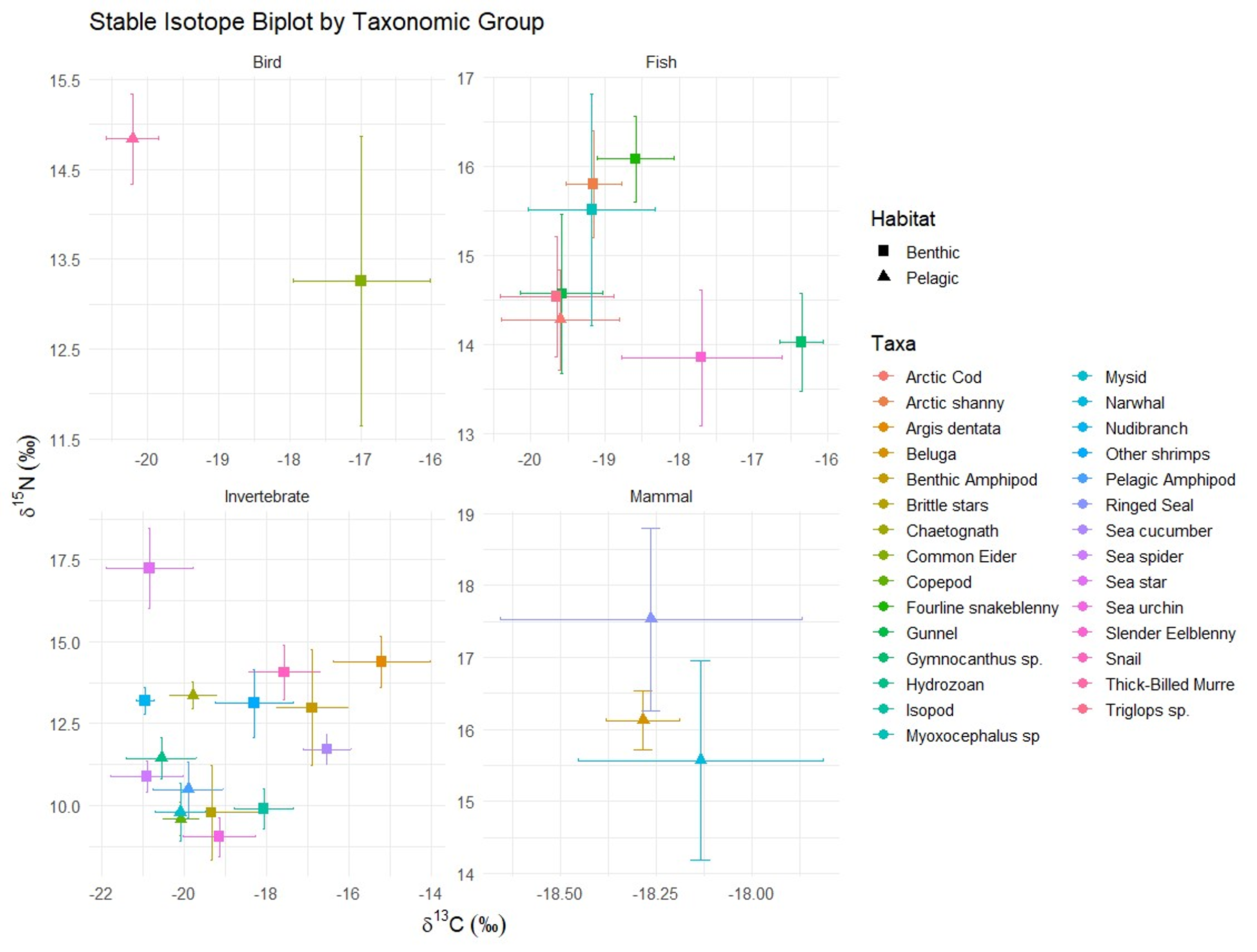
**

Figure S15. Stable isotope biplot (mean ± SD) based on δ¹³C and δ¹⁵N for benthic (squares) and pelagic (triangles) species by taxonomic group (birds, fish, invertebrates, and marine mammals).**
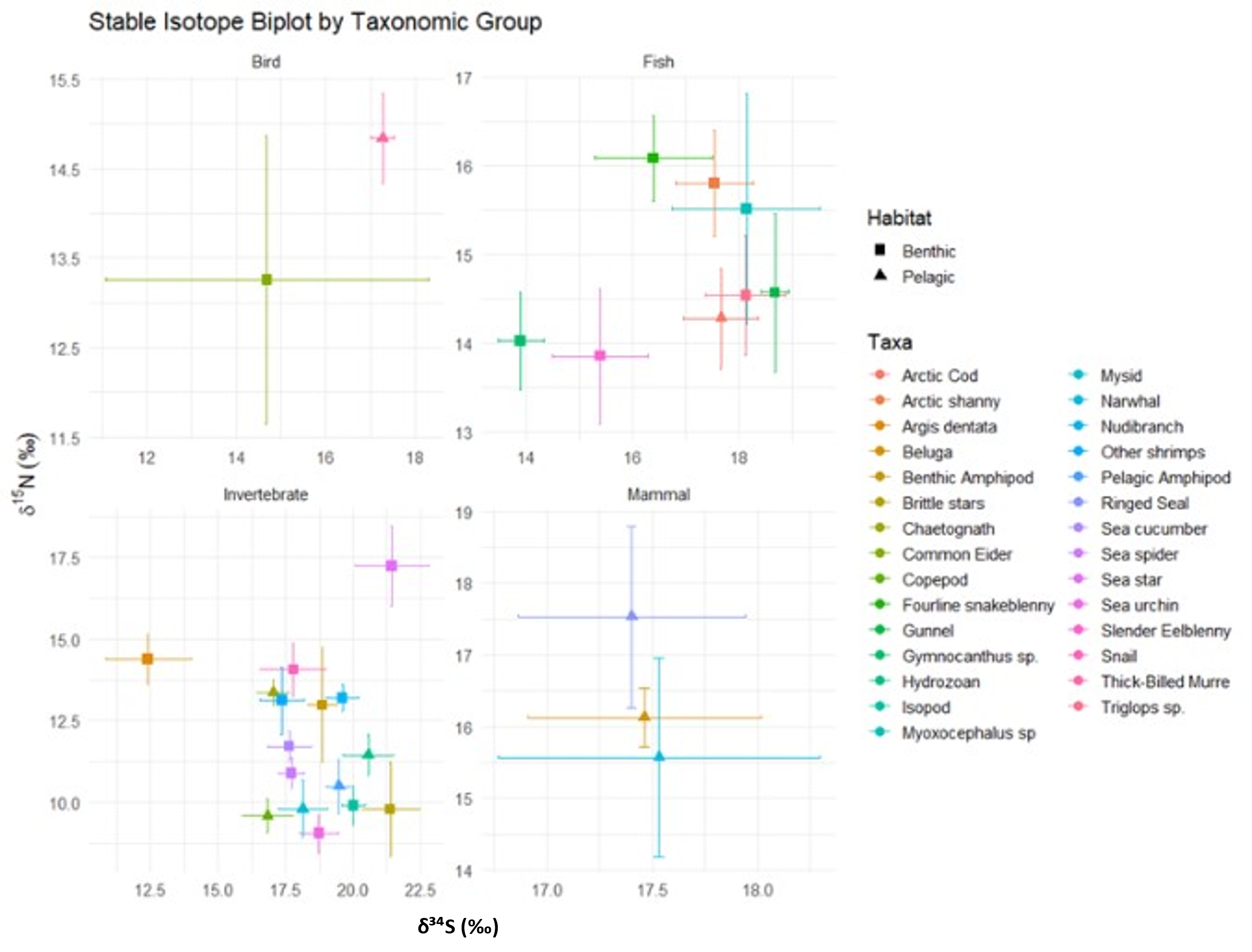
**

Figure S16. Stable isotope biplot (mean ± SD) based on δ³⁴S and δ¹⁵N for benthic (squares) and pelagic (triangles) species by taxonomic group (birds, fish, invertebrates, and marine mammals).

**
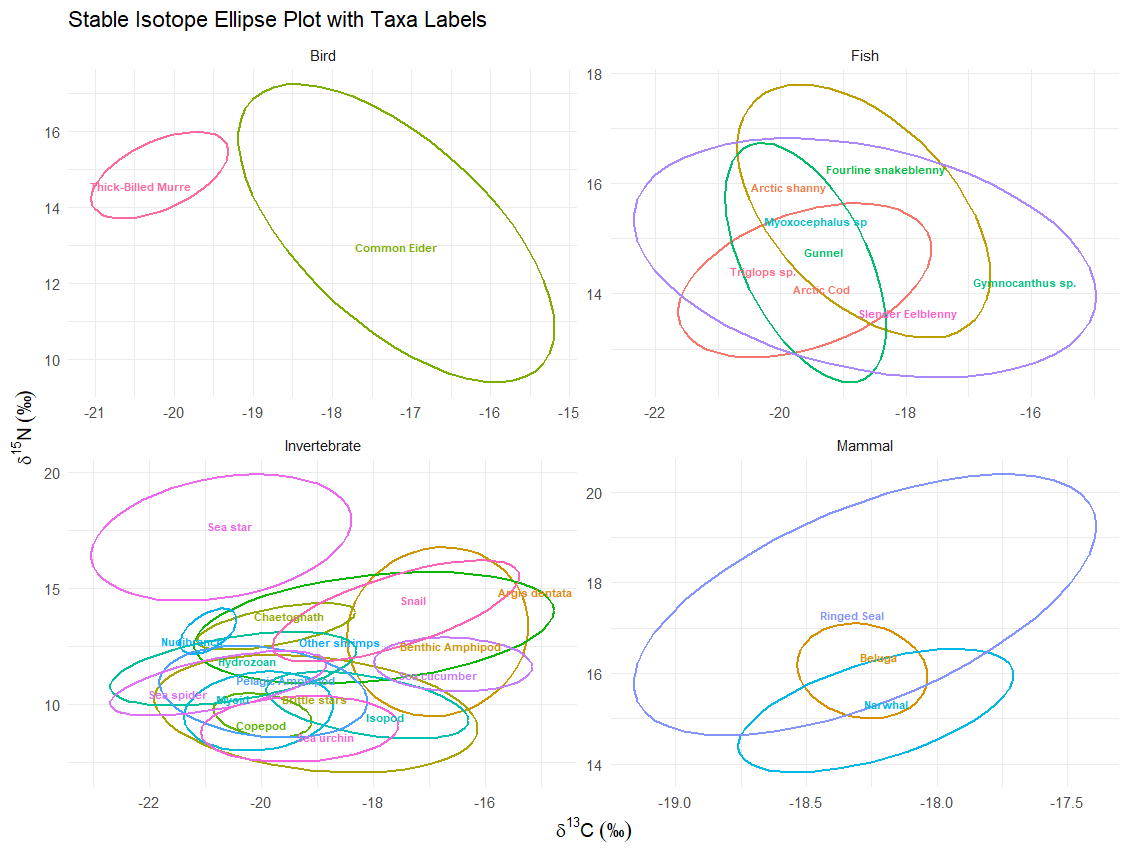
**

Figure S17. Bayesian standard ellipse areas (95%) derived from δ¹³C and δ¹⁵N for each taxon by taxonomic group (birds, fish, invertebrates, and marine mammals).

**
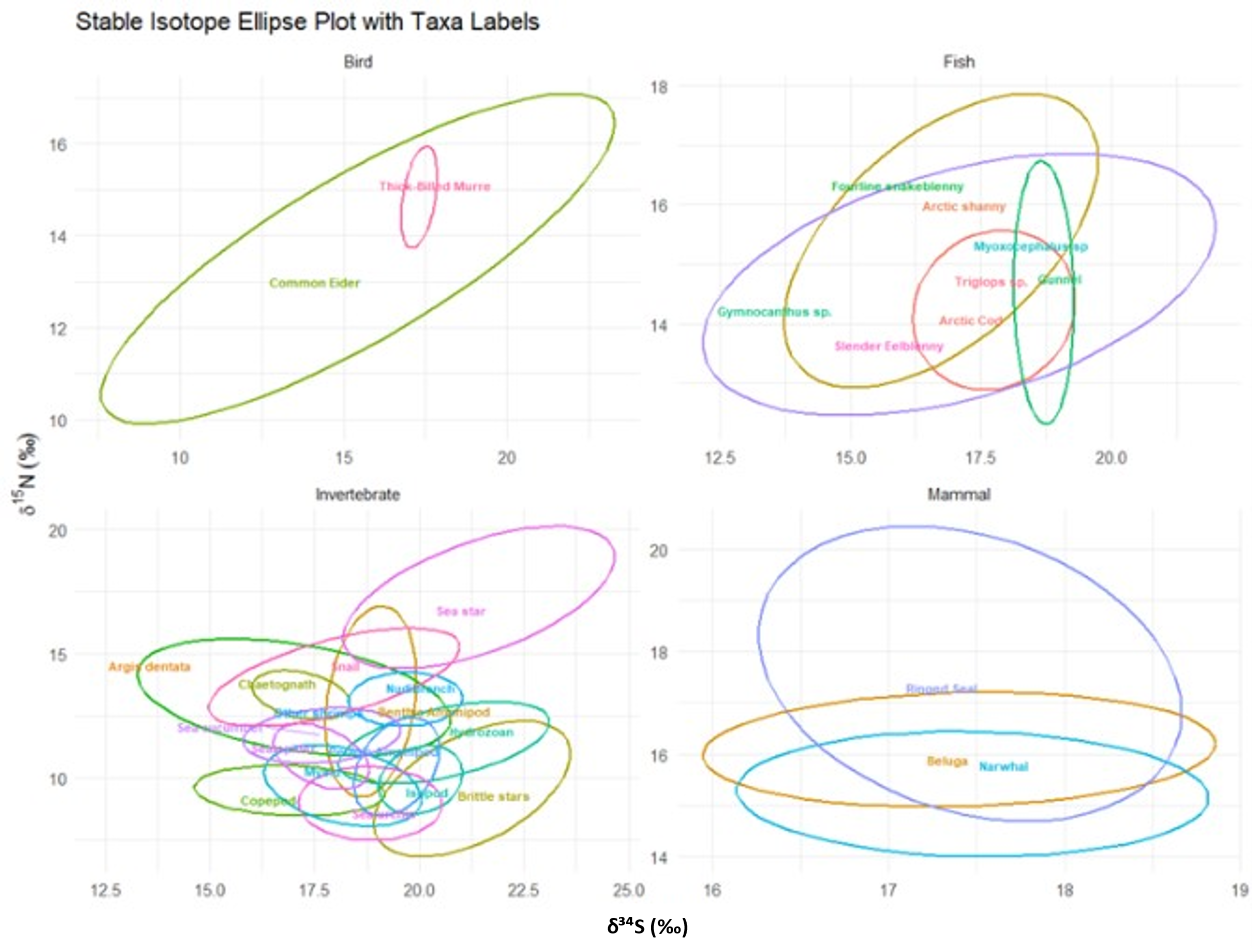
**

Figure S18. Bayesian standard ellipse areas (95%) derived from δ³⁴S and δ¹⁵N for each taxon by taxonomic group (birds, fish, invertebrates, and marine mammals).
